# Neurocomputational Mechanisms of Sense of Agency: Literature Review for Integrating Predictive Coding and Adaptive Control in Human–Machine Interfaces

**DOI:** 10.3390/brainsci15040396

**Published:** 2025-04-14

**Authors:** Anirban Dutta

**Affiliations:** Department of Metabolism and Systems Science, School of Medical Sciences, College of Medicine and Health, University of Birmingham, Birmingham B15 2TT, UK; a.dutta.1@bham.ac.uk; Tel.: +44-7395260645

**Keywords:** sense of agency, predictive coding, Kalman filter, active inference, SCAN, human–machine interface, functional neurological disorder, motor control, EEG microstates, LQG control, neurorehabilitation XR therapy, hypnotic suggestion

## Abstract

Background: The sense of agency (SoA)—the subjective experience of controlling one’s own actions and their consequences—is a fundamental aspect of human cognition, volition, and motor control. Understanding how the SoA arises and is disrupted in neuropsychiatric disorders has significant implications for human–machine interface (HMI) design for neurorehabilitation. Traditional cognitive models of agency often fail to capture its full complexity, especially in dynamic and uncertain environments. Objective: This review synthesizes computational models—particularly predictive coding, Bayesian inference, and optimal control theories—to provide a unified framework for understanding the SoA in both healthy and dysfunctional brains. It aims to demonstrate how these models can inform the design of adaptive HMIs and therapeutic tools by aligning with the brain’s own inference and control mechanisms. Methods: I reviewed the foundational and contemporary literature on predictive coding, Kalman filtering, the Linear–Quadratic–Gaussian (LQG) control framework, and active inference. I explored their integration with neurophysiological mechanisms, focusing on the somato-cognitive action network (SCAN) and its role in sensorimotor integration, intention encoding, and the judgment of agency. Case studies, simulations, and XR-based rehabilitation paradigms using robotic haptics were used to illustrate theoretical concepts. Results: The SoA emerges from hierarchical inference processes that combine top–down motor intentions with bottom–up sensory feedback. Predictive coding frameworks, especially when implemented via Kalman filters and LQG control, provide a mechanistic basis for modeling motor learning, error correction, and adaptive control. Disruptions in these inference processes underlie symptoms in disorders such as functional movement disorder. XR-based interventions using robotic interfaces can restore the SoA by modulating sensory precision and motor predictions through adaptive feedback and suggestion. Computer simulations demonstrate how internal models, and hypnotic suggestions influence state estimation, motor execution, and the recovery of agency. Conclusions: Predictive coding and active inference offer a powerful computational framework for understanding and enhancing the SoA in health and disease. The SCAN system serves as a neural hub for integrating motor plans with cognitive and affective processes. Future work should explore the real-time modulation of agency via biofeedback, simulation, and SCAN-targeted non-invasive brain stimulation.

## 1. Introduction

How does the brain seamlessly integrate external feedback and internal action planning to learn the subjective feeling of controlling one’s own actions and their effects on the external world, i.e., the sense of agency (SoA), in uncertain environments? The SoA is a fundamental aspect of human cognition and volition [1,2]. While several cognitive models have been proposed to explain the SoA, including the comparator model [3] and multifactorial frameworks [4], a comprehensive neurocognitive synthesis has remained elusive. This is largely due to the SoA’s complex nature, encompassing both low-level sensorimotor processes and high-level reflective judgments, as well as inconsistencies in terminology and conceptual boundaries across studies. For the purposes of integrating predictive coding and adaptive control in human–machine interfaces (HMIs), I will define the SoA as the outcome of a dynamic, hierarchical inference process in which the brain integrates sensorimotor signals (bottom–up) with contextual priors (top–down) to estimate the probability that oneself is the causal agent of an action and its outcomes. This process unfolds along two partially dissociable levels: the feeling of agency (FoA)—an implicit, pre-reflective experience of control—and the judgment of agency (JoA)—an explicit, reflective attribution of agency [2,4,5], where I postulate that internal action simulation plays a key role.

Emerging evidence highlights that disturbances in the SoA are a shared feature across a range of brain disorders—including schizophrenia, obsessive–compulsive disorder, autism spectrum disorders, corticobasal syndrome, functional neurological disorders—yet current treatment approaches lack specificity due to an insufficient understanding of the underlying cognitive mechanisms [5]. To address this gap, Malik et al. [5] proposed the Agency in Brain Disorders Framework, a novel model that integrates bottom–up and top–down cues, organized along prospective and retrospective timelines, to elucidate the diverse manifestations of SoA impairments in neuropsychiatric conditions. This framework also introduces standardized nomenclature and emphasizes the interplay between the FoA and the JoA as distinct but interacting components of agency experience. I focus on the process of SoA learning that often involves balancing uncertainty in external feedback—such as sensory feedback or instructional cues—with uncertainty in internal action simulation for preparatory action planning. In everyday life, the SoA underpins activities from driving a car to using a smartphone, where the brain uses internal action simulation to attribute outcomes to one’s own actions in complex human–technology interactions [6]. In HMIs—such as brain–computer interfaces, prosthetic limbs, or assistive automation—preserving and enhancing the SoA is vital so that users feel “I did that” rather than “the system did that”. Achieving this requires integration of neuroscience theory and engineering design, ensuring that HMIs align with the brain’s predictive processes [7]. This literature review provides an overview of computation models as they relate to brain–body–behavior analysis. I first outline the principles of predictive coding, Bayesian brain theories, and active inference. I then discuss how these frameworks explain SoA mechanisms and survey their application in HMI design—from seminal theoretical works to recent advances—focusing on peer-reviewed studies and computer simulations.

## 2. Computational Models and Neurophysiological Mechanisms

Predictive coding is a prominent framework in cognitive and computational neuroscience that views the brain as a Bayesian inference machine, constantly generating predictions about sensory inputs and updating its internal models based on prediction errors—the difference between the expected and actual input [8]. This theory suggests that perception, cognition, and action arise from the brain’s effort to minimize uncertainty by aligning top–down predictions with bottom–up sensory evidence. This section traces the evolution of predictive coding from early theoretical foundations to advanced computational and hierarchical Bayesian models, particularly in the context of perception–action coupling that subserves the SoA. Emphasizing theoretical and simulation-based approaches, it outlines key model architectures and assumptions and provides a comprehensive summary of major predictive coding models [9].

The historical development of predictive coding traces back to Helmholtz’s 19th-century idea of perception as unconscious inference [10], later echoed by Gregory’s view of perception as hypothesis testing [8]. These early ideas laid the foundation for a Bayesian view of brain function, where perception combines prior expectations with sensory evidence. Barlow’s principle of efficient coding introduced the notion that neurons should encode only unexpected information, aligning with predictive coding’s focus on transmitting prediction errors [8]. In the 1980s, empirical models like Srinivasan et al.’s retinal circuit showed how neurons could subtract predicted input, reducing redundancy [11]. In the 1990s, Mumford proposed a cortical architecture where top–down predictions meet bottom–up errors, and machine learning models like the Helmholtz Machine illustrated similar principles in unsupervised learning [8]. A major advance came with Rao and Ballard’s hierarchical model of predictive coding in vision, explaining neural responses through prediction-error signaling [12]. By the early 2000s, these ideas coalesced into the “Bayesian brain” hypothesis, establishing predictive coding as a central theory for perception and broader brain function [8]. Here, predictive coding was recognized as a key algorithmic motif for implementing such Bayes-optimal perception [13]. Chris Frith and colleagues introduced the comparator model of the SoA in the early 2000s [3]. In their paper [3], Frith et al. proposed the experience of control through two internal comparison processes that operate on different aspects of action monitoring. One comparator compares the desired state—the internally represented goal of an action—with the actual state informed by sensory feedback. When these two states match, the system generates a JoA. In contrast, another comparator compares the actual sensory consequences of an action with the predictions by the forward model based on the efference copy. A match between the actual and predicted sensory consequences leads to the FoA. Together, these two comparator processes integrate sensorimotor and predictive information to shape both the prospective and retrospective dimensions of the SoA. This work built on motor control theories but was seminal in applying computational motor prediction to self-awareness and agency in both health and neuropsychiatric conditions. Table 1 summarizes the significant predictive coding models, highlighting their key features and applications. Despite differences in implementation, they share a common thread: the use of prediction and error signals to drive information processing in a way that aligns with Bayesian inference [8].

Hierarchical generative models allow higher brain regions to predict activity in lower sensory areas, while only unexpected residuals (prediction errors) propagate upward—this framework has deep roots in the Helmholtzian idea of the brain as inferring causes of sensations [23], updated in modern form as the free-energy principle [18]. Early models like that of Srinivasan et al. [11] demonstrated predictive coding in the retina by showing how neurons transmit only residuals between actual and predicted inputs, reducing redundancy. This principle extended to hierarchical models from Mumford [14,15] to Rao and Ballard [16] in the visual cortex, where higher cortical areas predict lower-level activity, and only error signals are passed forward. Later refinements by Spratling introduced more biologically plausible mechanisms, integrating attention and nonlinear dynamics [9]. In the 2000s, Friston’s free-energy framework expanded predictive coding into a general theory of brain function, modeling cortical inference as minimizing variational free energy and incorporating anatomical constraints [18]. This evolved into active inference, unifying perception and action by treating behavior as a process of minimizing the expected prediction error, with applications in motor control, robotics, and social cognition [19,24,25]. The core assumptions included that every cortical area encodes a probability distribution over hidden causes (not just point estimates) [19], and neural dynamics perform a gradient descent on free energy, which is equivalent to Bayes-optimal perception [8]. More recently, deep learning models like PredNet [20] and Active Predictive Coding (APC) [22] have merged predictive coding with modern AI, enabling complex tasks like video prediction, part–whole learning, and hierarchical planning, demonstrating the scalability and generality of the predictive coding framework across neuroscience and artificial intelligence. APC represents a convergence of ideas: it draws from the original Rao–Ballard scheme [16], the active inference idea of policy selection by the expected free energy [24,26], and deep learning methods for function approximation [20]. This reflects a broader trend in the field to integrate predictive processing theories with advanced computational tools, to tackle complex behavior and cognition. Several models offered complementary insights, e.g., the forward and comparator models explain agency through motor command prediction and sensory feedback comparison [27]. The comparator model posits that agency arises when predicted outcomes match actual feedback [3]. Then, the free-energy principle [18] extended predictive coding by proposing that the brain minimizes surprise (free energy) to maintain equilibrium. The sense of agency arises from successful prediction–action alignment. The embodied cognition and Enactivism models [28,29,30] argue that agency emerges through real-time interaction with the environment—not internal models alone—i.e., agency is enacted, not inferred. The Dynamical Systems Theory model describes behavior as the emergent product of a self-organizing, multicomponent system evolving over time [31] which has been applied to understand human behavior, suggesting that agency arises from the self-organization of neural dynamics [32]. Ecological psychology [33], grounded in the work of James and Eleanor Gibson, offers a non-representational, action-oriented framework for perception and cognition, emphasizing the direct perception of affordances in the environment through dynamic agent–environment interactions.

My literature review within the context of design of HMIs using a Bayesian cue integration model of the SoA reframes agency as a cognitive inference about causality, rather than a purely sensorimotor phenomenon. In this model, the brain integrates bottom–up sensory signals (e.g., proprioception, action–effect timing) with top–down beliefs (e.g., prior expectations, intentions) to compute the probability that one’s action caused an outcome [34,35]. Each cue is weighted by its reliability or precision, allowing the brain to make a trial-by-trial inference about self-causation. This probabilistic approach explains how the SoA can vary across contexts, tasks, and individuals and accounts for its disruption in brain disorders [5]. Table 1 outlines five major predictive coding accounts of agency, showing how they implement the brain’s hypothesized ability to perform probabilistic inference by minimizing prediction error. Here, the exact Bayesian inference is computationally impractical for the brain due to the complexity of real-world generative models. Instead, it is proposed that the brain uses predictive coding as an approximate inference method, and this approach is grounded in the variational free-energy principle, where the brain does not apply Bayes’ rule directly. Instead, it aims to minimize a cost function (free energy), which includes a term based on Kullback–Leibler divergence [17]. This allows the brain to approximate posterior beliefs efficiently by reducing the difference between the predicted sensory input and the actual input, rather than computing exact probabilities. Implemented through hierarchical message passing (prediction and error units), this framework offers a neurophysiologically plausible account of perception, learning, and motor control that is reflected in cortical responses [17].

Early anatomical studies suggested that the primary motor cortex (M1) was not an isolated “motor strip” but included structural bridges (“plis de passage”) linking motor and sensory areas [36]. Although Penfield’s famous motor homunculus depicted the M1 as a continuous, body-mapped strip, even his own stimulations sometimes evoked complex sensations or urges rather than direct movements, hinting at a more integrative role [37]. Later studies in primates revealed that the M1’s somatotopy is overlapping and fragmented, with body part representations intermingled rather than neatly separated. This led to the idea that the M1 supports complex, coordinated actions through functional groupings and horizontal connectivity. These insights laid the groundwork for identifying the somato-cognitive action network (SCAN)—a set of integrative motor regions that link bodily control with cognitive processes like intention and agency [38]. The SCAN consists of three distinct cortical nodes located within the motor cortex, interrupting the traditional motor homunculus. These inter-effector regions lie between the classic zones for the foot, hand, and mouth along the precentral gyrus, forming an alternating pattern: foot–SCAN–hand–SCAN–face–SCAN. Rather than a smooth map, SCAN nodes intersperse the effector areas and are highly connected to higher-order brain networks—a key “nexus between body and mind” due to their integrative role. Anatomically, SCAN regions align with the classic “plis de passage” described by Gratiolet and Broca [39], now confirmed through MRI and cadaver studies as transverse gyri bridging motor and sensory cortices. These nodes blur the M1–S1 boundary and show distinct features: a thinner cortex, higher intracortical myelin, and unique connectivity patterns, including local and interhemispheric tracts. Found in both hemispheres and across species—including macaques and human infants—the SCAN appears to be a conserved and specialized part of the primate motor system.

In principal accordance, it is postulated that descending signals from the motor cortex resemble the feedback (modulatory) projections in sensory hierarchies rather than classic feedforward commands [40]. This leads to the active inference reinterpretation of motor commands—that the brain issues motor instructions by sending predicted proprioceptive signals to the muscles, and reflex arcs then fulfill these predictions. In other words, instead of commanding a muscle directly, the brain predicts the sensory state (e.g., of body, limb position) it wants, and the body responds to minimize the error. This perspective casts even basic reflexes as mechanisms to cancel out prediction errors, unifying perception and action under one predictive control scheme [40]. Such neural implementations make predictive coding a powerful model for understanding brain–body–behavior relationships. It explains phenomena like sensory attenuation: when we produce a movement, the brain’s prediction of the expected sensory consequences (via an internal forward model) attenuates the actual sensation [41]. For instance, self-generated touch or sounds feel less intense than external ones because the brain anticipated them. If the actual feedback deviates from prediction (e.g., a delay or perturbation is introduced), the prediction error triggers surprise and a change in perception—the basis for why one cannot tickle oneself effectively. This mechanism, present in healthy brains, is disrupted in in functional movement disorder (FMD), where it is postulated that aberrant top–down predictions—shaped by factors such as heightened self-focused attention, symptom expectations, and personal beliefs—can dominate and override actual sensory feedback [42]. In schizophrenia [43], hallucinations and delusions may arise from distinct predictive coding disruptions: hallucinations result from overly strong top–down predictions, causing the brain to ignore sensory input and perceive what it expects [44]; in contrast, delusions stem from weak priors and the excessive weighting of sensory evidence, leading to exaggerated prediction errors and false belief formation [45]. The review paper [43] argues that mismatch negativity can serve as a translational biomarker for detecting and differentiating predictive coding abnormalities, potentially guiding personalized interventions for such heterogeneity.

## 3. SCAN Mechanisms of Sense of Agency

SCAN regions stand out from surrounding motor areas due to their broad and integrative connectivity [36]. Unlike classic primary motor cortex (M1) effector zones (e.g., hand or foot areas), which are mostly limited to contralateral M1 and local S1, SCAN nodes connect extensively across both hemispheres and to multiple brain networks involved in action, cognition, and autonomic regulation. They are especially linked to the cingulo-opercular network (involved in attention and arousal), the SMA (efferent signaling [46]), insula, prefrontal cortex, basal ganglia, thalamus, cerebellum, and even autonomic centers like the adrenal medulla. The SCAN appears to integrate motor commands with the internal state, sensory feedback, and goal-directed behavior. Functionally, they co-activate during complex, multi-limb, or posture-related tasks, and neurosurgical evidence shows that the stimulation of SCAN nodes elicits diffuse, multi-effector responses. These regions act as flexible coordination hubs, linking isolated limb control with whole-body movement, intention, and context that is postulated to subserve the SoA that arises when our brain attributes an action and its contextual outcome to our own intent. The SCAN’s strong links to cognitive and proprioceptive networks suggest it plays a key role in generating movement intentions and comparing them with sensory feedback [47]. The stimulation of SCAN regions can evoke the illusion or urge to move, highlighting its involvement in the conscious experience of agency. The SCAN may integrate intent (from prefrontal/ACC), execution (M1), and feedback (S1, cerebellum), forming the basis of the feeling “I did that”. Disruptions in this integration could underlie disorders like alien limb syndrome, positioning the SCAN as a potential neural correlate of the conscious motor self.

**Figure 1:**
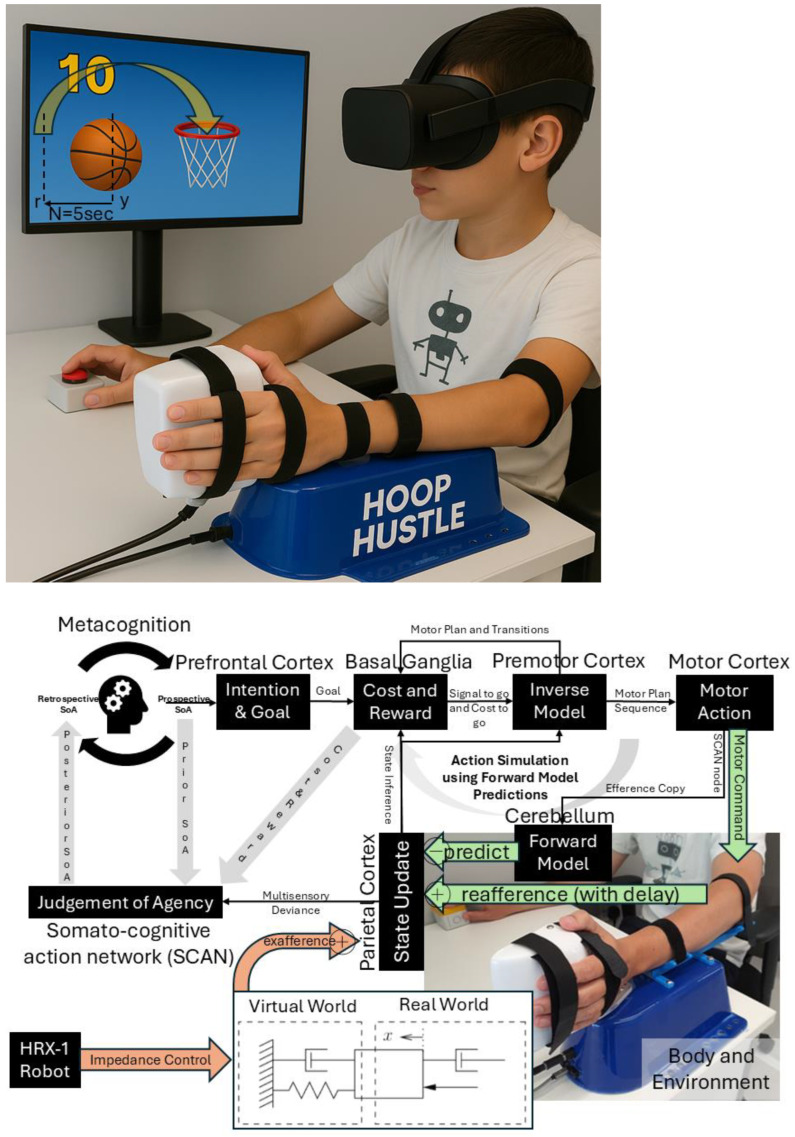
The top panel depicts the Hoop Hustle game, where players use an open-palm handle to guide a ball (y) toward a target (r) within a set time window (N) to initiate a shot and earn rewards in the form of visual and auditory feedback. The bottom panel illustrates the flow of information in a comparator model for motor control and agency. A motor command (efference copy) is sent from the motor system to the effector and simultaneously to a forward model, which predicts the expected sensory consequences of the action. The actual sensory feedback from the movement is then compared with the predicted outcome. One comparator (SCAN) matches the intended goal of an action with the actual outcome, producing a judgment of agency. Another (cerebellum—parietal cortex) compares the actual sensory feedback with predicted outcomes based on the efference copy, leading to the feeling of agency. Together, these processes combine sensorimotor and predictive information to form the sense of agency (SoA).

Classic theories in cognitive neuroscience have long postulated that the SoA critically depends on predictive mechanisms. The influential comparator model (see Figure 1) suggests that when we plan (inverse model) a voluntary action, an efference copy of the motor command is used by a forward model to predict the expected sensory consequences [6]. This predicted outcome (e.g., the sight, sound, or feel of the action’s effect) is then compared to the actual sensory feedback. If the actual outcome matches the prediction, the brain registers that “I caused this”, reinforcing the SoA. If there is a mismatch—for instance, the outcome is delayed, deviated, or differs in an unexpected way—a discrepancy signal arises, and the SoA is reduced or even abolished. Empirical evidence for this comparator mechanism comes from studies showing that altering feedback can modulate agency. For example, introducing temporal or spatial offsets between a person’s movement and its visual/auditory effect reduces the person’s feeling of control over that effect. Similarly, as noted, people cannot tickle themselves because the somatosensory consequences of a self-generated movement are predicted and hence attenuated; if that prediction is disrupted (e.g., by introducing an unexpected delay or using a robot arm to self-stimulate with altered feedback, i.e., with exafference [48]), the self-produced touch can feel more ticklish and less self-caused. These observations align with predictive coding: the SoA is strongest when the prediction error is minimal (expected and actual align), and it diminishes as the prediction error increases. Consistent with this, the predictability of outcomes has been shown to directly shape the agency. One view holds that agency is mostly inferred retrospectively—after action—based on how well the outcome matches the motor system’s intent and predictions. That is, the brain “decides” one was the agent if the sensory evidence fits the forecast of one’s own action effects, i.e., from the comparator mechanisms. However, more recent research indicates prospective cues also play a role: the fluency or ease of selecting and initiating an action can influence the SoA even before outcomes are known. In other words, when an action feels effortful or unusually constrained, people report lower agency, independent of the eventual outcome. Neuroimaging studies support this by linking feelings of agency to activity in premotor and parietal regions during action selection, not just outcome comparison, emphasizing the role of the prospective cues [49,50] that can be delivered with exafference for rehabilitation [46,51]. Here, predictive coding provides a computational design framework in which the brain’s top–down expectations as initial conditions and bottom–up sensory evidence interact to guide perception and action [52].

## 4. Predictive Coding Framework: Preparatory Activity Bayesian Prior

Predictive coding is closely tied to broader Bayesian brain theories, which posit that the brain encodes probabilistic beliefs and optimally combines prior expectations with sensory evidence [53]. Perception optimizes internal states to explain the world, whereas action optimizes external states (the world/body) to match internal predictions. The concept of perception–action coupling via predictive coding has deep connections to earlier ideas in motor control. For example, the “forward model” in motor control theory [54]—which predicts sensory consequences of one’s own actions—can be seen as a special case of the brain’s generative model, and the “inverse model” (computing motor commands to achieve desired outcomes) aligns with action-based prediction-error minimization. Active inference unifies these by positing that inverse models may not need to be computed explicitly; instead, if the brain holds a forward model (predicting sensory input given motor commands), then simply setting a desired outcome (sensory goal) and suppressing updates to that prediction (beta-band oscillations signal the “status quo” [55]) effectively recruits reflex arcs to make the prediction come true [40]. Indeed, Adams, Shipp, and Friston [40] demonstrated that classical motor reflexes (like maintaining posture) can be derived from active inference principles by “clamping” certain predictions and letting the motor plant reduce the error. Therefore, in active inference theory, perception and action are two sides of the same coin: perception optimizes internal predictions to fit sensory input, while action changes the external input to better fit the internal predictions [40]. In other words, if a mismatch exists between what the brain predicts and what it senses, it can either update its belief or act to make reality more like the prediction—this dual strategy means the brain’s control of the body is fundamentally model-driven. As noted by Friston et al., higher-level cortical areas send descending proprioceptive predictions (desired limb positions) instead of explicit motor commands, and the motor plant reacts reflexively to reduce the resulting error [40]. This aligns with the Equilibrium Point Hypothesis (EPH), introduced by Anatol Feldman [56], which posits that the central nervous system controls movements by setting desired equilibrium positions (desired limb positions) for muscles, allowing the inherent properties of muscles and reflexes to drive the limb toward these positions without specifying exact trajectories. This suggests that movement disorders (e.g., FMD, Parkinson’s) may arise from the disrupted predictive tuning of equilibrium points, leading to abnormal motor execution [57,58,59]. Here, active inference extends the EPH by adding a predictive dimension, allowing the nervous system to update equilibrium points dynamically. Through this lens, classical notions of reward or utility maximization are reformulated as inference: actions are chosen based on prior beliefs about achieving preferred outcomes [60], rather than directly maximizing reward [24]. For example, if the weather appears windy and rainy, prior beliefs about staying dry lead one to carry and hold an umbrella tightly, setting initial conditions (likely at the SCAN nodes of M1) for action based on expectations. Similarly, in the brain, decision-making can be viewed as a dynamical system, where initial neural states (shaped by prior experience) combine with incoming sensory inputs to drive evolving neural activity [52]. These findings suggest that decisions and adjustments emerge from the interaction between the initial conditions and sensory evidence, supporting a dynamical systems view of neural decision-making. The effectiveness of this adjustment depends on the brain’s preparatory and continuous estimation of sensory precision, akin to the Kalman gain in control theory, which determines the weight assigned to new sensory information versus prior beliefs [61]. However, this precision estimation is not always optimal [41]. While the predictive coding framework posits that prediction errors should diminish with repeated exposure (Kalman gain decreases—the system learns, and habituation occurs), persistent sensory responses in conditions such as Fragile X Syndrome challenge this view [56]. A possible reconciliation involves the impaired estimation of precision (possibly due to impaired neuromodulatory control), where sensory inputs are persistently overweighted—Kalman gain stays abnormally high, meaning the system keeps treating sensory input as surprising, even when it should be expected. Then, in conditions like FND, the brain, instead of updating beliefs in response to sensory feedback, may attempt to force “reality” to conform to the predictions [41]. This maladaptive strategy reinforces learned helplessness in FND. In summary, active inference provides a unifying framework: it not only accounts for perception as hierarchical Bayesian inference (akin to predictive coding) but also embeds the selection of actions into the same inferential process in health and disease [24]. This has practical implications for HMI design and robotics [46,48]. By harnessing the brain’s natural mechanisms of predicting sensory consequences and correcting errors, these HMI systems can facilitate human interactions while also serving as a rehabilitation tool by modifying rewards and sensory feedback to drive operant conditioning and adaptive learning [62]. Here, motor control involves two key components of agency: body awareness—the “Brain as Observer”, likely supported by SCAN regions—and action regulation—the “Brain as Controller”, likely handled by non-SCAN effector areas of the M1.

**Figure 2:**
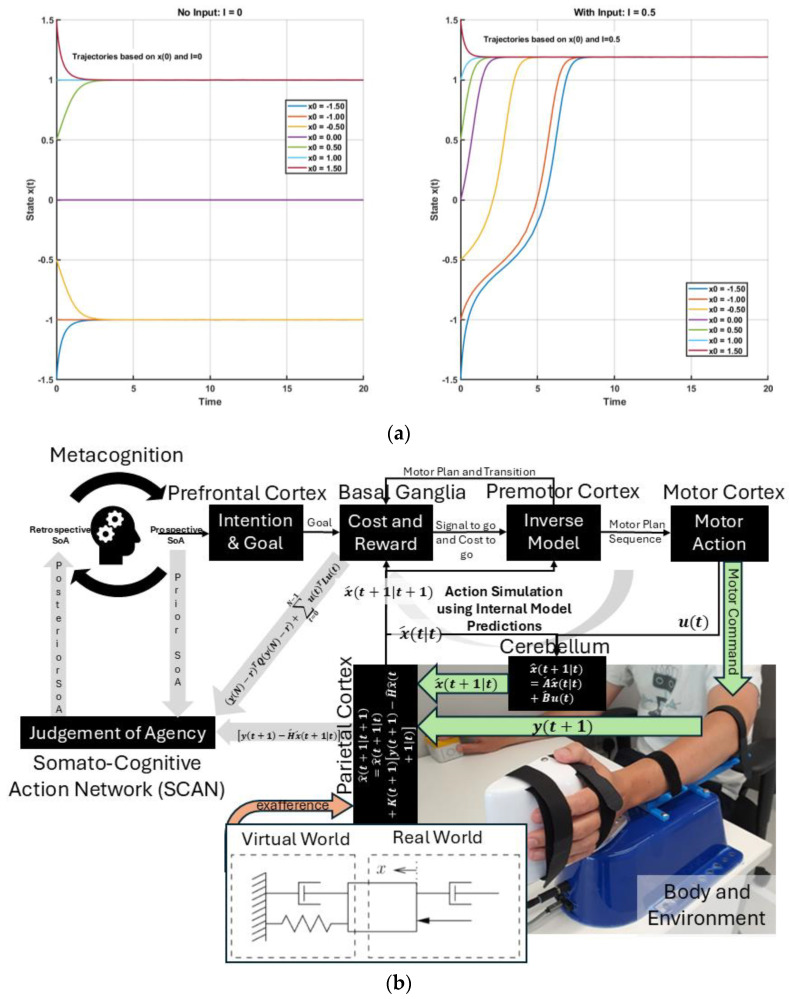
(**a**) This figure illustrates how initial conditions (x(0) = x0) and small biases in input (I) can lead to distinct long-term behaviors, relevant to decision-related neural dynamics. (**b**) Neurocomputational framework of agency and motor control in an XR environment, illustrating interactions across cortical and subcortical regions for intention, prediction, error monitoring, and metacognitive judgment using internal models, sensory feedback, and real-time haptic feedback integration.

Figure 1 shows how the interaction between the basal ganglia and premotor cortex supports motor planning by preparing the primary motor cortex in an optimal initial state. This includes setting body posture, arousal, and limb impedance for environmental interaction. Figure 2a shows an example of how initial conditions critically shape outcomes in a dynamical system. The differential equation for state, x: $\dot{x}=\mu x-x^{3}+I$, is integrated over time with the input, I. When I=0, the system is symmetric and unstable at zero, settling into either $+\sqrt{\mu }\;\mathrm{or}-\sqrt{\mu }$ depending on the starting point. When I≠0 (e.g., I=0.5), the system is biased toward one outcome. Simulating trajectories from different initial values (−1.5 to 1.5) showed that small differences in the starting state led to different long-term behaviors, analogous to how state can evolve into distinct decisions. In uncertain environments, effective behavior relies on balancing prior expectations with sensory feedback—essentially performing Bayesian inference [63,64]. Ample behavioral evidence supports this: humans combine cues (visual, proprioceptive, etc.) in a statistically optimal (Bayes-like) manner in tasks ranging from depth perception to motor adaptation. Under linear and Gaussian assumptions, classic results show that neural integration can approach the Bayes-optimal weighting of information (as in the Kalman filter model of sensorimotor integration [63,65]). Bayesian predictive coding merges these ideas by suggesting that the brain’s generative model encodes a probability distribution and prediction errors correspond to the updating of posterior beliefs. In fact, predictive coding has been described as a particular implementation of Bayesian inference—one way the brain might achieve approximate Bayes optimality through hierarchical error minimization [13]—and combining them through continual prediction-error minimization creates a simpler and more efficient approach following Occam’s Razor [66,67]. Active inference extends these concepts further by incorporating action into the prediction-error minimization loop.

## 5. Kalman–Bucy Filter and Linear–Quadratic–Gaussian Control

Active inference extends the principles of predictive coding by integrating perception and action into a unified framework that minimizes variational free energy [26]. Here, Kalman filters, widely applied in both engineering and neuroscience, offer a mathematically simple yet powerful framework for modeling dynamic sensorimotor integration. The Kalman filter operates in discrete time, updating state estimates at specific intervals, while the Kalman–Bucy filter works in continuous time, providing real-time updates via differential equations [61]. Discrete-time Kalman filters are commonly used in neuroscience because neural data are sampled in small time steps, making them intuitive for modeling brain–behavior dynamics. They help explain how the brain combines noisy sensory inputs with prior beliefs and internal simulations to optimize perception and learning [65]. These mechanisms are crucial not only for skill acquisition but also for clinical interventions, making Kalman filters valuable tools for understanding learning in complex, uncertain environments [63]. Despite their usefulness in modeling brain processes related to perception and action [13], Kalman filters have notable limitations, particularly their reliance on assumptions such as linearity (system’s dynamics and observation models are linear), Gaussian noise (both process noise and observation noise), and stationarity (state transition matrix, observation matrix, noise covariances) [68]. While predictive coding alone maps onto Kalman filtering—estimating hidden states through the minimization of prediction error in a Gaussian, linear system—active inference adds action selection by incorporating expected outcomes and control costs, thereby resembling Linear–Quadratic–Gaussian (LQG) control [69]. In this expanded view, Kalman filtering handles perceptual inference, while LQG-like optimization governs goal-directed behavior. Crucially, active inference does not treat perception and control as separate processes; instead, both are viewed as coupled components of free-energy minimization, where actions are chosen to fulfill predictions generated by internal models. This explicit integration—absent in traditional predictive coding—means that active inference embodies the combined logic of the Kalman filter and LQG control under a single Bayesian variational principle [69].

Consider the scenario in Figure 1 where “Hoop Hustle” is an XR therapeutic game designed for upper arm rehabilitation [51] using the HRX-1 haptic robot. The HRX-1 is a compact robotic interface developed by HumanRobotiX London for upper-limb neuromotor research. It provides the controlled actuation of wrist or elbow movements in both horizontal and vertical planes, with a ±140-degree range of motion and peak torque of 4 Nm. Weighing 4 kg, it features angle and torque sensing capabilities and offers flexible configurations, including wrist, elbow, and bimanual setups. The HRX-1 supports current, velocity, and position control modes and is programmable via MATLAB (R2023b), Simulink, and C/C++. Additionally, it integrates with EMG and EEG devices for comprehensive neuromechanical analysis. Participants used their affected arm, using the palm handle (see Figure 1) to shoot virtual balls through hoops positioned at varying locations. They received real-time visual feedback via a Meta Quest 3 headset with immediate visual and auditory rewards for successful scores. The XR game adapts the difficulty (e.g., virtual haptic spring–damper) based on individual ability. Players must move the open-palm handle (see Figure 1) to move the ball (y) to a target (r) within a set time (N) to shoot and earn rewards (visual/auditory feedback). Figure 2b builds on Figure 1 by illustrating the dual roles of feedforward action simulation and delayed reafferent feedback in motor control and agency. Motor planning begins in the prefrontal cortex with intention setting, followed by basal ganglia evaluation of cost and reward to select the optimal motor plan via internal inverse models. This plan is issued through the supplementary motor area (SMA) to the SCAN node of M1 for preparatory activity and simultaneously sent as an efference copy to forward models in the cerebellum. When the body is ready for action with preparatory activity, a signal to go is delivered to the non-SCAN effector nodes of M1. Predicted sensory outcomes are compared with actual feedback in the parietal cortex, with any mismatch used to update beliefs and decipher the judgment of agency. The “Virtual World” haptic spring–damper system (see Figure 2b) is controlled via a Kalman filter and LQG controller in humans.

The task objective can be formally described as minimizing a quadratic cost function that captures both reward-based positional accuracy and body energy expenditure:

$$J={\left(y\left(N\right)-r\right)}^{T}Q\left(y\left(N\right)-r\right)+\sum_{t=0}^{N-1}{u\left(t\right)}^{T}Lu\left(t\right)\;$$

Here, y(N) is the ball position at final time N; r is the target location; Q is a matrix defining the positional error cost at the final time (t=N); u(t) denotes motor commands at time t; and L represents the cost associated with motor commands. The relative magnitude of Q and L indicates the trade-off between error cost and effort—this may subserve comorbid fatigue in FND [70]. Here, the effort can be modulated using a haptic spring–damper, as shown in Figure 1, to modulate human trade-off between error cost and effort.

To successfully complete the task, participants require an internal model, i.e., a predictive relationship between motor commands and their sensory outcomes (e.g., proprioceptive and visual states). This “Hoop Hustle” is a novel task for the participants, so this must be learned [71]. We represent the state-space model dynamics linearly as follows:

State prediction: $\hat{x}(t+1|t)=\hat{A}\hat{x}(t|t)+\hat{B}u(t)$;

Sensory feedback prediction: $\hat{y}(t)=\hat{H}\hat{x}(t|t)$.

Here, $\hat{x}\left(t|t\right)$ is the predicted state (e.g., body and ball) at time *t* given actual sensory feedback up to that time; $\hat{A}$, $\hat{B}$, and $\hat{H}$ are learned (adapted over time through experience) matrices defining how states evolve without input (e.g., physics of arm dynamics) ($\hat{A}$ is the state transition matrix), how motor commands (e.g., muscle activations) affect the states ($\hat{B}$ is the control matrix), and how internal states map to sensory feedback (e.g., vision, proprioception) translating into their prediction, $\hat{y}(t)$ ($\hat{H}$ is the observation matrix), where $\hat{x}\left(t+1|t\right)$ is the predicted state at time *t* + 1 given the predicted state, $\hat{x}\left(t|t\right)$, and motor command, u(t), at time *t*. The controllability matrix is $C=[B\;AB\;A^{2}B\;\cdots A^{n-1}B]$, and the observability matrix is $O=\left[\begin{array}{c} H\\ HA\\ \begin{array}{c} HA^{2}\\ \vdots \\ HA^{n-1} \end{array} \end{array}\right]$, where *n* is the number of state variables. The state estimate update (correction) at time *t* + 1 given new sensory feedback at *t* + 1, $y\left(t+1\right)$, will be $\hat{x}\left(t+1|t+1\right)=\hat{x}\left(t+1|t\right)+K\left(t+1\right)\left[y\left(t+1\right)-\hat{H}\hat{x}(t+1|t)\right]$. The mixing gain matrix, $K\left(t\right)$, determines how much the state estimate should be corrected based on prediction errors (balances between prediction and measurement), which may not be optimal (Kalman gain) in FND [41]. Figure 2 expands on Figure 1, highlighting the parallel roles of feedforward simulation (SCAN nodes of M1) and delayed reafferent feedback in motor control (non-SCAN effector nodes of M1). Figure 1 shows how the prefrontal cortex initiates intentions and supports the metacognitive evaluation of agency, using the prospective sense of agency to guide preparatory motor activity before action at M1 [72]. The basal ganglia evaluates a combination of body cost and reward by minimizing an objective function of the form, (${\left(y\left(N\right)-r\right)}^{T}Q\left(y\left(N\right)-r\right)+\sum_{t=0}^{N-1}{u\left(t\right)}^{T}Lu\left(t\right)$), using internal inverse models (decision-related dynamics at premotor cortex [52]), selecting the optimal motor plan that minimizes this cost till the final time (*t* = *N*). Once the best motor plan is selected and the body is ready for action following preparatory activity, the basal ganglia delivers a “Go” signal to initiate the corresponding motor plan sequence till the final time (*t* = *N*). The SMA complex receives the selected motor plan by upstream evaluative systems (e.g., basal ganglia–dorsal premotor cortex interactions) that is issued to the non-SCAN effector nodes of M1 for execution and is simultaneously broadcast by SMA with a SCAN node of M1 as an efference copy to internal forward models (cerebellum) [46]. This efference copy serves as an input to a predictive model—often formalized as part of a Kalman filter state estimator—which generates an internal prediction of the movement’s sensory consequences. These predictions are then compared against actual sensory feedback to compute a prediction error, which is used for state update, i.e., update (parietal cortex) about the system’s state (e.g., body and environment). Here, I postulate that SCAN serves as a key computational hub in the judgment of agency—see Figure 2b. The prediction error reflects the mismatch between the predicted and actual sensory feedback SCAN (dorsal anterior cingulate cortex plays a key role) and is integrated with additional contextual variables such as the final cost and reward (from basal ganglia) associated with the action and the prior belief about agency held before the action—together, these signals shape a posterior SoA.

Interaction with the body and environment—including exafference via a haptic spring–damper system (HRX-1 robot [51])—enables perturbations using exafference to probe goal-directed movement and provide operant conditioning (by modulating effort, L) in dysfunctions like those seen in FND [51]. Given these estimates, the control problem (subserved by the basal ganglia ↔ premotor cortex loop) reduces to finding the motor commands ($u\left(t\right)$) that minimize the expected future costs (“cost-to-go” (Riccati) equation) [71], $u\left(t\right)=-G\left(t\right)\hat{x}\left(t\right)$, where $G\left(t\right)$ is the feedback gain at time t, computed recursively backward via a dynamic programming algorithm that solves the underlying LQG control problem [73] (optimality assumed in healthy individuals [74]), i.e.,

$$G\left(t\right)={\left(L+B^{T}W\left(t+1\right)B\right)}^{-1}B^{T}W\left(t+1\right)A\;$$

$$W\left(t\right)=A^{T}W\left(t+1\right)\left[A-BG\left(t\right)\right],\;\;\;W(t=N)=H^{T}QH$$

The feedback gain ($G\left(t\right)$) encodes how beliefs about states map into motor commands, effectively defining the optimal control policy based on backward recursion (Riccati equation). Here, movement elements are consolidated (e.g., motor chunking [75]) with state estimation computed forward in time and optimal control computed backward to guide action. In FND, the control policy may not be optimal [41], inhibiting the action.

In practice, motor commands $u\left(t\right)$ introduce noise, $\varepsilon_{u}\left(t\right)$, which can be modeled as zero-mean Gaussian noise whose variance is proportional to the magnitude of the control signal itself, $\sum_{i}C_{i}u(t)\varphi_{i}(t)$, where $\varphi_{i}(t)∼N(0,1)$ is an independent standard Gaussian random variable. Thus, the full stochastic dynamics of the internal model are as follows:

$$\hat{x}\left(t+1|t\right)=\hat{A}\hat{x}\left(t|t\right)+\hat{B}\left(u\left(t\right)+\sum_{i}C_{i}u(t)\varphi_{i}(t)\right)+\varepsilon_{x}\left(t\right),\;\;\;{\;\varepsilon }_{x}\left(t\right)∼N\left(0,X\right)$$

$$\hat{y}\left(t\right)=\hat{H}\left(\hat{x}\left(t|t\right)+\sum_{i}D_{i}x\left(t|t\right)\mu_{i}\left(t\right)\right)+\varepsilon_{y}\left(t\right),\;\;\;\varepsilon_{y}∼N\left(0,Y\right)$$

$${\;\;\varphi }_{i}\left(t\right),\mu_{i}\left(t\right)∼N\left(0,1\right)$$

where *ε_x_* and $\varepsilon_{y}$ denote additive Gaussian noise in state evolution and sensory observations, respectively, with covariance matrices *X* and *Y*. During XR game play, sensory measurements *y*(*t*) are continuously integrated with model predictions $\hat{y}\left(t\right)$ to update beliefs about the state.

$$W_{x}\left(N\right)=Q\left(N\right)$$

$$W_{e}\left(N\right)=0$$

$$W_{x}\left(t\right)=Q\left(t\right)+A^{T}W_{x}\left(t+1\right)\left[A-BG\left(t\right)\right]+\sum_{i}{D_{i}}^{T}H^{T}K^{T}(t)A^{T}W_{e}\left(t+1\right)AK(t)HD_{i}$$

$$W_{e}\left(t\right)={\left(A-AK(t)H\right)}^{T}W_{e}\left(t+1\right)\left[A-AK\left(t\right)H\right]+A^{T}W_{x}(t+1)BG(t)$$

$$G\left(t\right)={\left(L(t)+\sum_{i}{C_{i}}^{T}B^{T}W_{x}\left(t+1\right)BC_{i}+\sum_{i}{C_{i}}^{T}B^{T}W_{e}\left(t+1\right)BC_{i}+B^{T}W_{x}\left(t+1\right)B\right)}^{-1}{\;B}^{T}W_{x}\left(t+1\right)A$$

**Figure 3:**
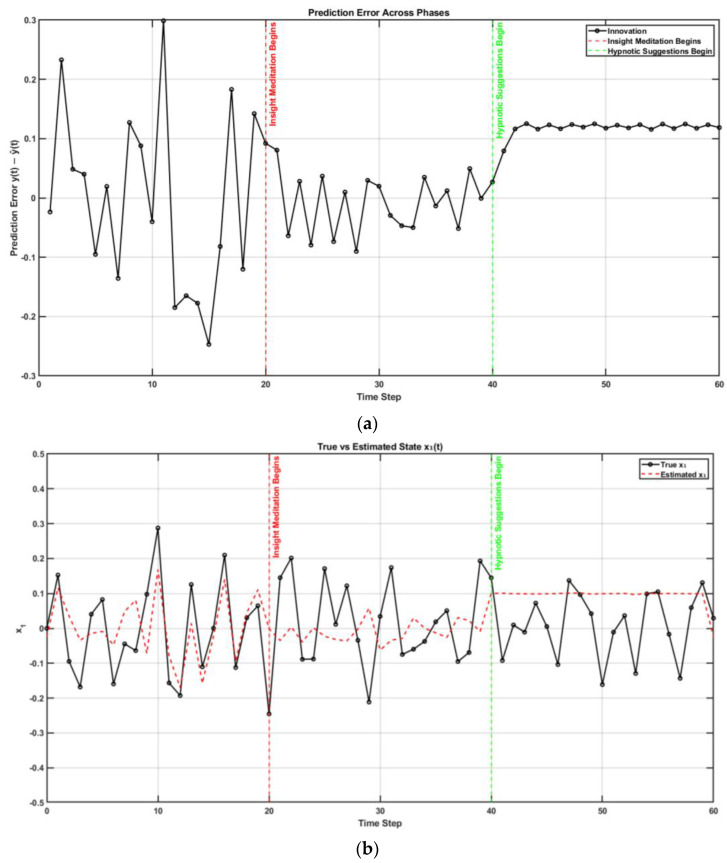
Visualization of belief updating under Insight Meditation and hypnotic suggestion: (**a**) prediction error plot and (**b**) true vs. estimated position over time, illustrating how suggestion biases the equilibrium setpoint, driving limb movement without conscious intent.

In control theory, two key properties—controllability and observability—determine whether a system can be effectively regulated and monitored. A system is controllable if it can be driven from any initial state to a desired one through control inputs (evaluated via the controllability matrix). It is observable if its internal states can be reconstructed from external outputs (determined through the observability matrix). These dual properties are foundational in designing adaptive and stable systems, such as in robotics [68]. Extending this framework, I propose that the judgment of agency depends on the perceived controllability and observability of one’s body and environment, learned through a metacognitive Ladder of Causation. When observability is reduced (e.g., due to societal opacity), or controllability is undermined (e.g., by coercion), one’s sense of agency can deteriorate. Mental simulation can serve as a representational tool for understanding both the self and others [76] through a metacognitive Ladder of Causation. As illustrated in Figure 2b, mental imagery can engage action simulation via the forward model, which can be shaped by external suggestions (exafference) [77]. Hypnotic suggestion (considered exafference), often defined as a process that enhances the realism of imagined scenarios, can shape perceptions of body ownership, agency, and size [78]. Hypnotic suggestions are typically preceded by hypnotic induction techniques, such as focused breathing and heightened bodily awareness [78] as preparatory activities. In this state of “focused attention and reduced peripheral awareness, characterized by an enhanced response to suggestion” [79], reafference is minimized and suggestions are trusted as the sole sensory input (exafference), $S_{ext}$, shaping state expectations $\hat{x}\left(t+1|t+1\right)=\hat{x}\left(t+1|t\right)+K\left(t+1\right)\left[S_{ext}-\hat{H}\hat{x}(t+1|t)\right]$. Then, mental imagery, as a form of enactment imagination [80], relies on sensory visualization and shares neural mechanisms with perception. Mental imagery functions as an internal simulation system, using forward models to predict the outcomes of potential actions and guide belief updating [41]. This process supports adaptive learning and problem-solving by enabling the brain to simulate future scenarios without physical movement. In the context of Insight Meditation, there is no external sensory feedback; instead, the mind primarily engages with internal noise ($y\left(t+1\right)∼N\left(0,V\right)$) [41]. Through consistent meditation practice, the variance in this internal noise (V) is reduced, enhancing the state expectations $\hat{x}\left(t+1|t+1\right)=\hat{x}\left(t+1|t\right)+K\left(t+1\right)\left[y\left(t+1\right)-\hat{H}\hat{x}(t+1|t)\right]$ [41]. Figure 3 shows a MATLAB (Mathworks Inc. Natick, Massachusetts, USA) simulation of a three-phase cognitive control process using the LQG control framework to model how full attention on all sensory inputs, internal simulation without sensory input (in Insight Meditation [41]), and hypnotic suggestions without sensory input affect state estimation and motor behavior (code, figure3.m at https://github.com/Siagnos/ICRA2025 accessed on 10 April 2025). It simulates a 10 Hz oscillatory system (physiological oscillations ~10 Hz) under feedback control, with state estimates updated via a Kalman filter. In Phase 1 (*t* = 1–20), the system has access to all sensory inputs (reafference) and uses a Kalman filter for the internal state estimate—representing a condition with full attention to sensory inputs. In Phase 2 (*t* = 21–40), the sensory input is no longer available, but the system continues to simulate internal dynamics with minimizing external noise, representing internal simulation in Insight Meditation [41] where belief updating is mostly driven by prior expectations. In Phase 3 (*t* = 41–60), the system incorporates external suggestions (bias term for exafference as sensory input), simulating how hypnotic suggestions can shape future expectations in the absence of real-world sensory input. Throughout, the code tracks the prediction error and applies LQR control using the estimated state. These results are visualized through two complementary figures: a prediction error plot (Figure 3a) and a comparison of the true versus estimated position over time (Figure 3b). Together, these plots illustrate how hypnotic suggestions dynamically modulate belief updating and motor control. Specifically, the persistence of steady-state prediction error under suggestions reflects a biased setpoint, which—according to the Equilibrium Point Hypothesis—can drive limb movement even in the absence of conscious motor intent. In line with these simulation results, I propose the integration of virtual reality (VR)-based hypnotic suggestion within a platform technology for eXtended Reality biofeedback training under operant conditioning (using suggestions) for FMD rehabilitation [81]. In this framework, brain imaging—particularly beta synchronization observed in the SMA during post-movement stages in individuals with FND—can provide a biomarker of sensorimotor integration (efferent copy, u(t)–Figure 2b) [46]. Prior research has demonstrated that the hypnotic state modulates sensorimotor beta rhythms during both real movement and motor imagery [82]. Hypnotic suggestion has been shown to alter motor cortex excitability [83] and modulate corticospinal output during motor imagery [84], highlighting its potential as a tool for reshaping dysfunctional motor representations [85] likely subserved by the SCAN nodes of M1. These findings open new avenues for applying cognitive neuroscience principles [77] to XR therapeutic interventions in FND.

**Figure 4:**
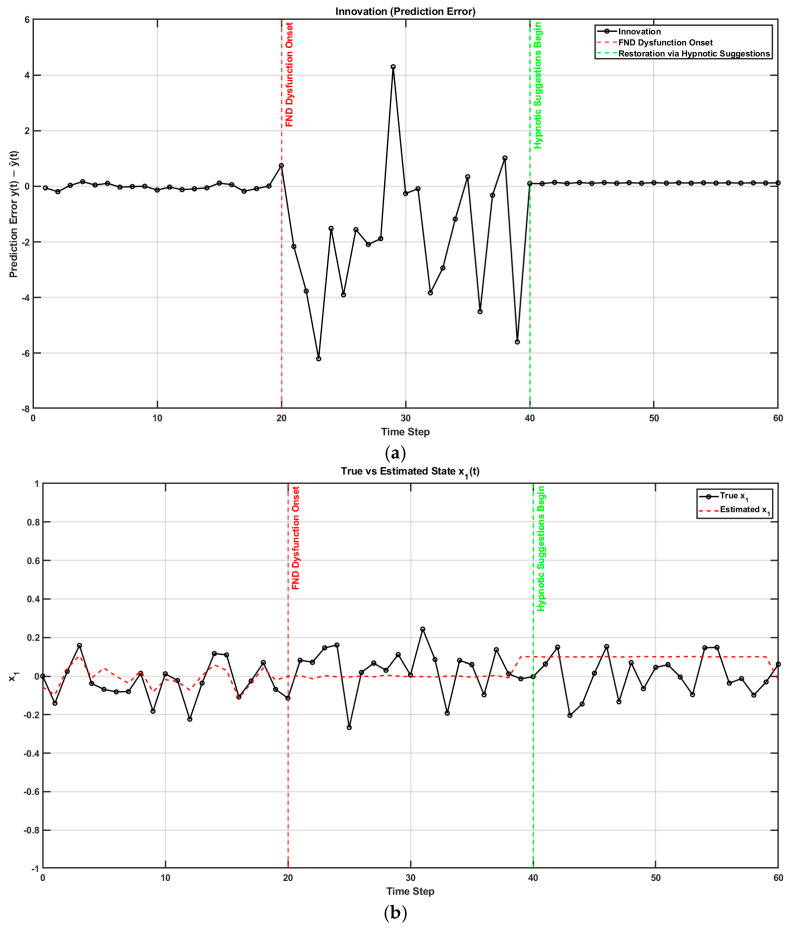
Visualization of belief updating under FND dysfunction and restoration by hypnotic suggestion: (**a**) prediction error plot and (**b**) true vs. estimated position over time, illustrating how suggestion recalibrates sensory-model balance, restoring disrupted inference in FND.

Figure 4 shows a MATLAB (Mathworks Inc. USA) simulation of how altered sensory integration in FND affects motor control [42,86] and how hypnotic suggestions may restore normal function (code, figure4.m at https://github.com/Siagnos/ICRA2025 accessed on 10 April 2025). Initially, the Kalman gain is optimal, allowing the accurate integration of sensory feedback and internal predictions. At *t* = 20, the Kalman gain becomes suboptimal, simulating FND-like dysfunction where the brain underweights sensory input (i.e., overconfidence in prior beliefs) [87]. This leads to growing prediction errors and poor state estimation. At *t* = 40, hypnotic suggestions are introduced by restoring sensory precision (focused attention with hypnotic induction), which increases Kalman gain and improves belief updating [41]. The observed reduction in prediction error (Figure 4a) and the convergence between estimated and true states (Figure 4b) demonstrate how hypnotic suggestions can recalibrate the balance between sensory evidence and internal models, effectively correcting the disrupted inference seen in FND. A reduction in the prediction error facilitates a posterior sense of agency with hypnotically suggested movements that feel involuntary and therefore effortless.

The Kalman filter and LQG control align with active inference, a framework in which the brain predicts the sensory outcomes of movements and minimizes discrepancies between expected and actual sensations by adjusting motor commands (inverse model) and/or updating predictions (forward model). Here, I propose that the brain can simulate alternative actions and outcomes to incorporate the Ladder of Causation [88], a framework introduced by Judea Pearl, which consists of three levels:

Association—recognizing correlations between variables without inferring causality, i.e., associative learning using a comparator model (prediction error = ‖expected outcome−observed outcome‖) [89].

Intervention—understanding the effort and impact of deliberate actions on outcomes, moving beyond passive observation, i.e., active inference (active minimization of variational free energy) [26] and causal inference [5].

Counterfactuals—engaging in retrospective revaluation [41], leading to assessing agency under ambiguity, i.e., counterfactual regret minimization [90].

Integrating retrospective revaluation enhances motor learning through action simulation via predictive coding and counterfactuals [4]. Activation in the right prefrontal cortex and ventral striatum was observed during retrospective revaluation [91], providing support. In the context of the free-energy principle [26], an agent evaluates possible actions by minimizing the expected free energy, which balances the predicted rewards (what it wants) and information gain (what it needs to learn) [26]. This approach unifies perception and decision-making, treating both as processes of reducing uncertainty and achieving goals. Within a retrospective revaluation framework, the brain retrospectively revaluates interventions—from action execution to action simulation—to discern for agency under ambiguity [91]. Counterfactual regret minimization (CFR) is a computational method used to approximate Nash equilibria in extensive-form games [90]. CFR is guaranteed to converge to a Nash equilibrium in two-player, zero-sum games with perfect recall, where players remember past decisions. When noise is introduced—reflecting real-world uncertainty—players may not settle into a fixed strategy but instead exhibit patterns consistent with a stochastic equilibrium, where strategies are stable on average. This approach offers a more realistic understanding of human decision-making under ambiguity [92] that is also relevant in skill learning.

## 6. “Brain as Controller” vis-à-vis “Brain as Observer” in Skill Learning

**Figure 5:**
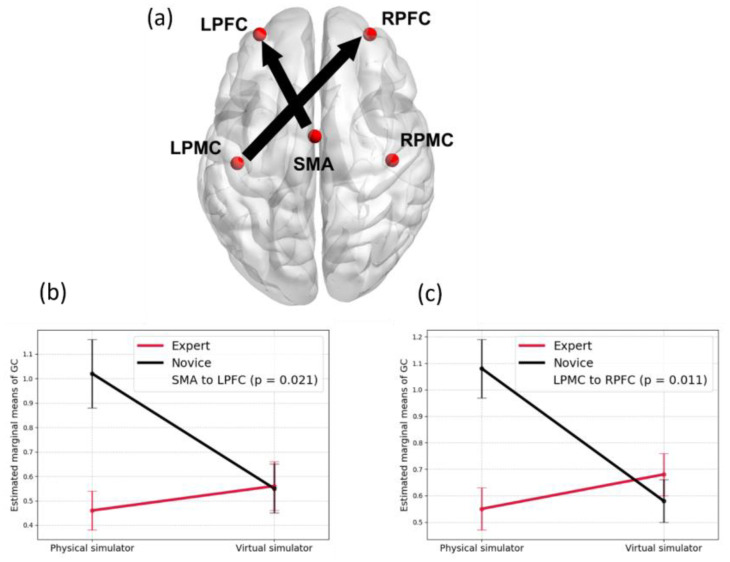
Interaction between expertise and simulator technology was observed, with (**a**) significantly differentiating connectivities from the supplementary motor area (SMA) to the left prefrontal cortex (LPFC) and from the left sensorimotor cortex (LPMC) to the right prefrontal cortex (RPFC)—regions implicated in motor planning and the retrospective evaluation of actions; (**b**) a significant interaction effect (*p* = 0.021) on SMA→LPFC connectivity; and (**c**) a significant interaction effect (*p* = 0.011) on LPMC→RPFC connectivity. Significant inter-regional directed functional connectivity (FDR = 0.05, Benjamini–Hochberg adjusted) is indicated by black arrows. Reproduced from Kamat et al. [93] under a Creative Commons Attribution 4.0 International License.

Skill learning with a medical simulator based on directed brain connectivity showed an interaction between expertise (experience level) and simulator quality (contextual realism) [93]. Figure 5 shows how directed brain connectivity is influenced by the interaction between expertise and simulator type during skill learning. In the context of laparoscopic surgical skill acquisition [93], the virtual simulator is novel for both groups, while the physical simulator is only novel to novices. The analysis reveals significant differences in the directed brain connectivity between groups, specifically from the supplementary motor area (SMA) to the left prefrontal cortex (LPFC) and from the left sensorimotor cortex (LPMC) to the right prefrontal cortex (RPFC)—regions implicated in motor planning and the retrospective revaluation of actions [91] likely subserved by the SCAN nodes of M1.

**Figure 6:**
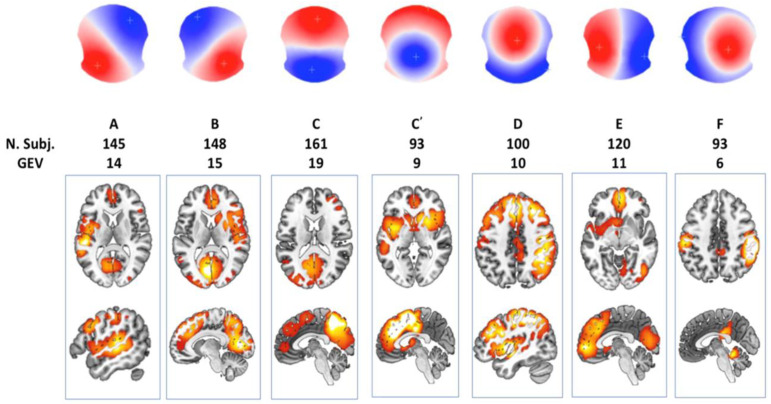
Source localization of seven EEG microstates was performed by correlating EEG microstate time courses with current densities estimated via a generalized linear model. GEV represents the global variance explained by each microstate. Figure reused from Michel and Koenig [96], under CC BY 4.0 license.

In this section, I examine brain activations, as described by Brechet et al. [94], mapping them onto the steps of the Kalman filter and LQG control framework introduced in Section 5. This analysis is conducted through a combined functional near-infrared spectroscopy (fNIRS) and electroencephalogram (EEG) microstate analysis from prior work [95]. Figure 6, adapted from a review by Michel and Koenig [96], illustrates canonical EEG microstates, each representing distinct brain activation patterns associated with unique and shared cortical regions. The classic four-state scheme (labeled A, B, C, and D) is presented, along with additional variants C′, E, and F. Microstates A and B represent diagonal opposites in spatial distribution (e.g., left anterior to right posterior for A and the reverse for B)—I postulate in “Brain as Observer” state. Microstates C and D exhibit strong anterior–posterior contrasts with inverted polarities—pattern C shows red frontal and blue occipital regions, while pattern D shows the opposite—I postulate in “Brain as Controller” state. Variant C′ features a central polarity surrounded by an oppositely charged ring, whereas E and F introduce lateral (left–right) and fronto-central asymmetries in datasets that exceed the four-class model. These spatial patterns help characterize which lobes (frontal vs. occipital) and hemispheres (left vs. right) exhibit primary positivity or negativity. Notably, the data—collected from 164 participants using a 256-channel EEG system—demonstrate that not all individuals express every microstate, particularly for patterns C and D, suggesting inter-individual variability (“Brain as Controller” in internal processing). This is further highlighted by the division of Microstate C into subtypes, reinforcing the non-uniformity of brain activation patterns.

**Figure 7:**
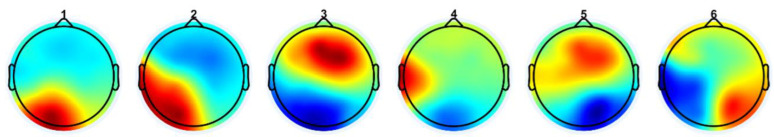
The six microstates identified through k-means cluster analysis of EEG-fNIRS time series across subjects during the “suturing and intracorporeal knot-tying” task in the Fundamentals of Laparoscopic Surgery (FLSs) program. Figure reused from Walia et al. [95], under CC BY 4.0 license.

Figure 7 presents task-related EEG-fNIRS microstate patterns from our own study [95]. Pattern 1 reveals inferior–posterior positivity and superior negativity. Pattern 2 exhibits a diagonal gradient with lower-right positivity and upper-left negativity. Pattern 3 emphasizes fronto-central positivity and posterior–inferior negativity. Pattern 4 features a left–right asymmetry, with a red hotspot over the left-central area and blue–green activity on the right. Pattern 5 reflects frontal or fronto-superior positivity versus posterior negativity. Pattern 6 displays a bilateral dipole, with the left and right hemispheres showing opposing polarities depending on the color scale. A qualitative comparison between these patterns and those from the review paper [96] suggests partial overlaps, though not exact one-to-one correspondences. Specifically, our MS patterns 1 and 3 show anterior–posterior polarity distributions, resembling the review MS patterns C or D, depending on the sign. MS pattern 2 corresponds to the diagonal structure of patterns A or B. MS patterns 4, 5, and 6 demonstrate lateral asymmetries, analogous to the review patterns E and F [95].

**Figure 8:**
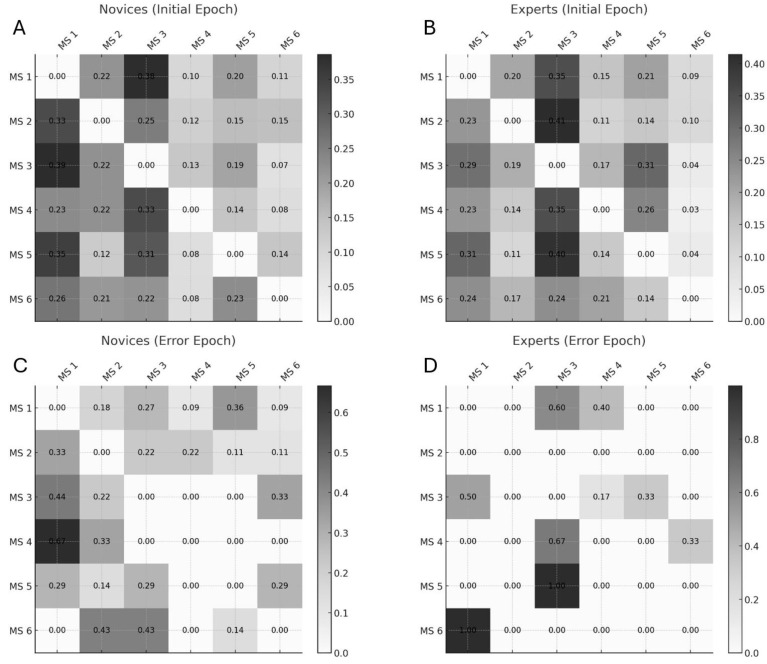
Transition probability statistics between microstate (MS) classes at the group level: (**A**) during the initial 10 s of the FLSs complex task in novices, (**B**) during the initial 10 s in experts, (**C**) during the 10 s error epoch in novices, and (**D**) during the 10 s error epoch in experts. Rows represent the “from” microstate, and columns represent the “to” microstate. Figure reused from Walia et al. [95], under CC BY 4.0 license.

The review paper [96] also distinguishes MS pattern C′ as a separate subtype, further segmenting the classic C pattern beyond the six-state models used in Walia et al. [95] and Brechet et al. [94]—this suggests a finer classification granularity. When comparing EEG microstates across studies, several methodological factors must be considered. EEG map signs are inherently arbitrary—for instance, a red-top/blue-bottom pattern is functionally identical to a blue-top/red-bottom one. This makes direct visual comparisons across studies potentially misleading. Additionally, differences in referencing schemes, such as using a common average versus average mastoids, can further alter the appearance of topographies by shifting or tilting the overall map. These factors highlight the importance of caution when interpreting or comparing EEG topographies purely based on visual features. Nevertheless, traditional EEG microstate analysis ignores topographical polarity, treating reversed patterns as the same state, which can miss important neural dynamics [97]. By accounting for polarity, Kashihara et al. [97] uncovered more detailed and continuous microstate transitions. They found that transitions typically occur within the same polarity, while between-polarity shifts are rare and structured. Notably, aging altered these patterns—older adults showed fewer transitions along key routes like D-C-E, more within-polarity shifts among A, D, and B, and an increase in uncommon transitions. Including polarity offers a clearer, more accurate view of brain activity over time. Moreover, the number of microstates modeled (e.g., 6 vs. 7) can impact classification outcomes, where additions like C′ reflect finer subtyping within established categories. To objectively evaluate the similarity between the review’s microstates (A–F) and our own (1–6), we compute Pearson correlation coefficients and mean squared errors (MSEs) between their spatial distributions, using normalized pixel values. This quantitative comparison provides an empirical basis for mapping and aligning microstates across studies. Transition probabilities between EEG microstate classes (see Figure 8) revealed distinct patterns differentiating experts from novices in their brain responses to task errors, as reported in Walia et al. [95]. These neural responses were mapped onto steps of the proposed Kalman filter model, wherein predicted internal states are adjusted based on sensory prediction errors—a process central to motor control and error correction.

A key mechanism underlying expert performance is implicit mental imagery, which refers to the unconscious activation of sensorimotor brain regions during motor tasks. Unlike explicit mental imagery, where individuals intentionally visualize movement, implicit imagery occurs automatically—often during motor planning or real-time error correction—and is especially prominent in expert performers [98,99]. In the study by Walia et al. [95], the expert group consisted of nine attending surgeons and residents with over one year of experience in laparoscopic surgery, while the novice group included thirteen medical students with no prior exposure to laparoscopic procedures. This contrast allowed for the examination of expertise-related neural signatures during task performance and error monitoring. Further extending this investigation, a separate study [99] involved a cohort of surgical trainees and professionals performing an explicit mental imagery task. In this task, participants—comprising junior residents (PGY 1–3, *n* = 10), senior residents (PGY 4–5, *n* = 10), and attending surgeons (*n* = 10)—were asked to dictate a simulated operative note, simulating the cognitive demands of intraoperative decision-making. During the surgical epoch of this dictation task, brain activity was recorded using fNIRS, focusing on the prefrontal, sensorimotor, and occipital regions. These data were used to analyze underlying brain states, offering insight into how different levels of surgical expertise manifest in distinct neural activation patterns during surgical tasks.

**Figure 9:**
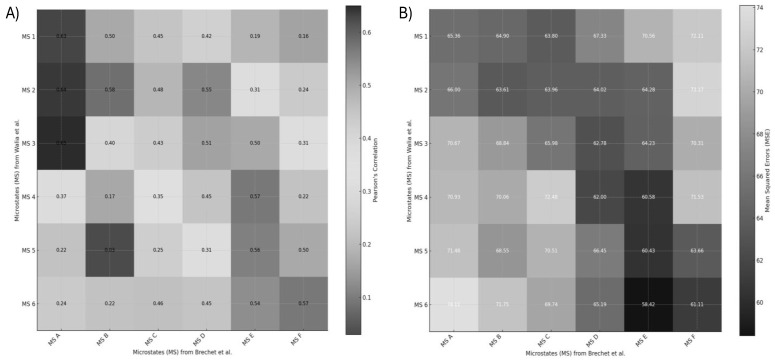
Similarity scores between the microstates from Walia et al. [95] and Brechet et al. [94], calculated using Pearson correlation coefficients (**A**) and mean squared errors (**B**), highlighting their differences.

Figure 9 presents the similarity scores between the EEG microstates identified in Walia et al. [95] and those from Brechet et al. [94]. Similarity was quantified using Pearson correlation coefficients and mean squared error (MSE) values. Higher Pearson coefficients and lower MSE values indicate stronger alignment between corresponding microstate topographies. The comparative analysis revealed several notable correspondences. In cases of one-to-many mappings, selection was guided by neurophysiological correlates described in both Walia et al. [95] and Brechet et al. [94].

Microstate 1 from Walia et al. aligns with a combination of Microstates A and C from Brechet et al., all showing an anterior–posterior gradient, albeit with slight differences in polarity and intensity.

Microstate 2 corresponds to a mix of Microstates A and B, characterized by lateralized brain activity.

Microstate 3 maps onto both Microstates A and D, sharing central dominance, though Microstate D exhibits a narrower spatial distribution.

Microstate 4 closely aligns with Microstate E, with both showing balanced anterior–posterior symmetry; Microstate E, however, displays a slight frontal skew.

Microstates 5 and 6 correspond to a combination of Microstates E and F, both reflecting left–right asymmetries.

Despite the well-established role of EEG microstates during resting state, their implication in the generation of motor behavior is debated [100]. Evidence of such a functional role of spontaneous brain activity would provide support for the design of novel and sensitive biomarkers not only for skill learning but also in neurological disorders [100]. Pirondini et al. [100] showed that the subject-specific microstates’ dynamical organization correlated with the activation of muscle synergies and can be used to decode individual grasping movements with high accuracy. These findings highlight the relevance of task-related microstate analysis over resting-state analysis [101] for revealing how spontaneous brain activity encodes motor control, offering potential for personalized biomarkers in skill learning and neurorehabilitation. The functional relevance of these microstates has been extensively characterized by Tarailis et al. [102], who linked each to distinct neural networks and cognitive functions. Microstate A is commonly associated with the auditory network, showing negative BOLD correlations in the bilateral superior and middle temporal gyri and sometimes in the medial prefrontal cortex (MPFC) and occipital cortex (OC)—suggesting a role in sensory integration. Microstate B corresponds to the visual network, marked by BOLD signals in the occipital cortex and strong activation in lateral and medial parietal areas, including the precuneus and retrosplenial cortex, which are involved in visual scene construction and episodic memory. Microstate C has been associated with both the salience network (SN) and default mode network (DMN) in different contexts. It shows positive BOLD activity in the dorsal anterior cingulate cortex (dACC), bilateral inferior frontal gyrus, and right insula—regions tied to salience detection and interoceptive awareness. Subtypes such as Microstate C′ have been identified in studies using higher-resolution decomposition (more than four microstates), showing nuanced variants of this core pattern. Microstate D is linked to the frontoparietal network (FPN) and attention systems, displaying BOLD activity in bilateral inferior frontal gyri, dACC, and superior parietal lobules/intraparietal sulci. This pattern supports cognitive control, attentional reorientation, and task switching. Notably, the dACC’s role also aligns it with the cingulo-opercular network (CON) and salience network, acting as a hub for dynamic network switching between the DMN and FPN [103]. Microstate E has been linked to the right medial prefrontal cortex (MPFC) and is thought to support intuitive, automatic decision-making and interoceptive processes, often aligning with the salience network. Microstate F involves bilateral MPFC activation, corresponding to the anterior default mode network (aDMN). It is associated with self-referential thinking, mental simulation, and the theory of mind. These functional mappings help explain the neurophysiological underpinnings of the similarities and subtle differences observed between the microstate patterns in Walia et al. [95] and Brechet et al. [94].

**Figure 10:**
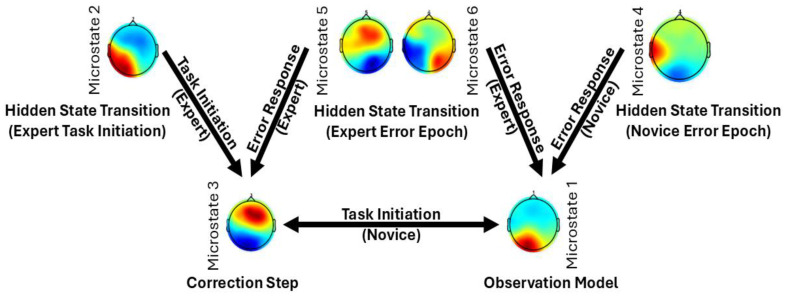
Interpretation of expert and novice groups’ microstate transitions based on the Kalman filter framework. Figure adapted from Walia et al. [95], under CC BY 4.0 license.

Figure 10 presents a Kalman filter framework interpretation of microstate transitions observed in expert and novice participants during the Fundamentals of Laparoscopic Surgery (FLSs) task. Figure 10 supports that EEG microstate transitions are not purely discrete but reflect continuous neural dynamics [97]. Continuous neural dynamics under the Kalman filter framework help to conceptualize how the brain updates internal models in response to sensory feedback and prediction errors.

### 6.1. Microstate Transitions During the Error Epoch

In the error epoch, novices primarily transition from Microstate 4 (corresponding to Microstate E) to Microstate 1 (a combination of Microstates A and C). This transition likely reflects a shift from automatic/intuitive processing (Microstate E) to an observation model involving sensory integration (Microstate A) and SN + DMN (Microstate C). Such patterns suggest a novice-level response to task errors, wherein conscious observation and sensory recalibration dominate. In contrast, experts show a more refined transition pattern. They begin in Microstate 6, which integrates features of Microstates E and F—reflecting both intuitive decision-making (right medial prefrontal cortex, MPFC; Microstate E) and deliberate cognitive control (left MPFC; Microstate F). This bilateral MPFC activity suggests the experts’ ability to balance automatic and controlled processes, effectively regulating error-related arousal before transitioning to Microstate 1, the observation model.

Another notable transition in experts occurs from Microstate 5 (aligned with Microstate E) to Microstate 3 (a combination of Microstates A and D). Here, Microstate D primarily represents the FPN, critical for the Correction Step [104] in the Kalman filter and LQG control framework. Within Microstate 3, sensory integration (Microstate A) and motor planning areas such as the DMFC—including the SMA—are thought to interact with the dACC from Microstate D. This interaction enables the transformation of error signals into corrective motor plans. Importantly, the dACC serves as a communication hub across multiple networks, the CON and the SN [105], both of which are implicated in goal-directed behavior and prediction-error signaling [106]. This makes Microstate D—involving the dACC, inferior frontal gyrus, and parietal regions—suited also for Bayesian belief updating in response to surprising or unexpected events [107].

In the context of FLSs learning, it is proposed that the CON uses inputs from the SN to detect salient events and then recruits the FPN to guide adaptive motor responses [108] by the effector nodes of M1. In the error epoch, Microstates 5 and 6 (experts) and Microstate 4 (novices) are likely involved in Hidden State Transitions (implicit mental imagery) in the Kalman filter model.

### 6.2. Microstate Dynamics During Task Initiation

During the initiation phase of the FLSs task, novices exhibit frequent transitions between Microstates 1 and 3, reflecting an interplay between the “Brain as Observer” and the “Brain as Controller”. Here, the SCAN involves the dACC, SMA, insula, and inter-effector M1 nodes—areas linked to Microstate C. Functionally, one may view the SCAN as coordinating global motor programs and mapping them onto the local executors (the limb-specific neurons). During an actual movement, SCAN nodes in M1 might help adjust posture, activate stabilizing muscles, modulate autonomic responses (e.g., raising heart rate via adrenal connection for a strenuous action), and ensure the movement aligns with the intended goal (via connections to cognitive control centers). This pattern aligns with the cognitive stage of skill learning [93,109], characterized by a high cognitive load and conscious performance monitoring. Experts, however, predominantly transition to Microstate 3, with strong transitions from Microstates 2 and 5. Here, Microstate 2 (a hybrid of Microstates A and B) involves sensory integration (A) and episodic memory recall via the precuneus/retrosplenial cortex (B). This suggests a more autonomous, experience-driven form of Hidden State Transition (implicit mental imagery), where implicit mental imagery supports the experts’ control mechanisms—characteristic of an autonomous stage.

### 6.3. Internal Simulation Model and Task Observation

In our Internal Simulation Model for enactment imagination, we propose that mental imagery facilitates the Correction Step by enabling individuals to simulate corrective actions internally for reevaluation (subserved by SMA, LPFC, LPMC, RPFC—see Figure 5). Here, the observation model reflects brain activity during first-person observation and varies with experience [109]. Enactment imagination is the mental rehearsal of movement without actual execution [110]. It engages the brain’s motor planning circuits, particularly regions that also activate during real movement—though at lower intensity. Studies show that imagined actions activate areas like the M1, premotor cortex, and SMA and suppress sensorimotor rhythms in EEG, similar to actual movement. The SCAN appears to play a central role in internal simulation by integrating intention, planning, and sensory prediction, without driving overt motion. SCAN regions in M1, with their high activation thresholds and links to cognitive control areas, likely allow us to “feel” the movement internally while keeping the body still. This is supported by findings that motor cortex excitability increases during imagery but stays sub-threshold—enabling rehearsal without action [111].

Functional imaging studies show that mental imagery recruits higher-order networks, such as CON and FPN circuits, more than execution does. SCAN nodes help maintain the imagined action through connections with the prefrontal, parietal, and cerebellar regions, supporting both the sense of agency and the internal simulation [41]. In this way, the SCAN provides the neural foundation for the vivid experience of movement in the mind’s eye, bridging cognition and motor control—see Table 2. Mental imagery engages distinct regions of the SCAN, each contributing to different aspects of simulated movement. The middle SCAN region (SCAN-M), located between the hand and mouth areas in M1, is strongly activated during bimanual, tool-related, and goal-directed imagery. It supports internal movement modeling and sequencing through its connections with the premotor cortex, inferior parietal lobule (IPL), and dorsolateral prefrontal cortex (dlPFC). The ventral SCAN region (SCAN-V), situated below the mouth area, is involved in the imagery of facial gestures, vocalizations, and emotionally charged actions, likely aiding interoceptive simulation and affective embodiment via links to the insula, orbitofrontal cortex (OFC), and anterior cingulate cortex (ACC). The dorsal SCAN region (SCAN-D), between the foot and hand zones, is less explored but may contribute to imagery involving posture, gait, or full-body coordination. Together, these regions facilitate the planning, prediction, and embodied experience of imagined actions. Therefore, SCAN brain regions participate in both real and imagined movements, but they take on an especially crucial integrative role during imagery when overt motor output is absent. Both conditions share the activation of these “somato-cognitive” nodes—reflecting overlapping neural substrates—yet the balance shifts: execution leans more on M1 effector-specific activation and sensory feedback, whereas imagery leans more on M1 SCAN-mediated internal modeling and cognitive network support. Our study [109] investigated how brain activation during a cognitive surgical task—simulated operative dictation (an internal simulation task)—varies with surgical experience. Using fNIRS, we measured brain activity across the prefrontal, sensorimotor, and occipital regions in junior residents, senior residents, and attending surgeons. The results showed that senior residents exhibited significantly greater activation in the left prefrontal, premotor, and parietal cortices compared to both juniors and attendings. These findings suggest that cognitive-task-related brain activation patterns differ by experience level and highlight the potential of functional neuroimaging as an objective tool for assessing cognitive surgical expertise.

## 7. Discussion

In this review paper, I applied the Kalman filter and LQG control framework to understand how brain activation during a cognitive–motor task varies with surgical experience, focusing on transitions between EEG microstates. During FLSs task initiation, novices showed high transition variability—especially between Microstates 1 (“Brain as Observer”) and 3 (“Brain as Controller”)—reflecting exploratory strategies typical of early learning. In contrast, experts demonstrated more efficient and focused transitions, primarily into Microstate 3 from Microstates 2 and 5, enabling controlled action through prediction-error-based processing [94,95,112]. During the error correction phase, experts used quick robust transitions (e.g., Microstate 5 to 3; Microstate 6 to 1) [113], while novices relied on more variable transitions. The observation model was supported by SCAN-linked microstates (A, C), involved in self-monitoring and memory retrieval [1,114], while the Correction Step engaged the FPN, SN, and CON via Microstates D and 3, facilitating adaptive control and behavior modification [108,115,116]. Enactment imagination further distinguished skill levels [99], with experts showing localized SMA activation—critical for motor intentionality and integration across the DMN, FPN, and sensorimotor systems [113,114,116,117]. Disruptions in these dynamics are observed in FMD, where abnormal beta synchronization, heightened self-focus, and impaired SN–FPN switching impair motor control [118,119,120], and motor imagery mechanisms may underlie their symptoms [121]. Neurological evidence suggests that the SCAN network plays a key role in linking intention with movement, contributing to the sense of agency—the feeling of “I am initiating this action”. Disruption to the SCAN may impair this link, as seen in patients with this motor pathway damage who struggle with motor imagery. In contrast, motor imagery training may strengthen SCAN connectivity when this pathway is intact. A recent study [122] also found that the SCAN connects many key neuromodulatory targets for Parkinson’s disease, including areas treated with deep brain stimulation. This highlights the SCAN as a central hub in motor control under the Kalman filter framework, see Table 3, potentially involved in disorders like Parkinson’s, where self-initiated movement is impaired and motor imagery remains relatively preserved. Individuals with aphantasia, who lack visual mental imagery, also have impaired motor simulations. A study by Dupont et al. [123] found that, unlike those with typical imagery, people with aphantasia did not show increased corticospinal excitability during motor imagery or action observation, indicating reduced motor system activation. However, it is still unclear whether SCAN regions are specifically affected in aphantasia, and further research will be key to deciphering SCAN involvement in cognitive–motor tasks.

Recent findings [124,125,126] support the view that sensorimotor performance, such as tracking tasks, depends on dynamic, embodied engagement with environmental feedback. Older adults exhibited reduced tracking accuracy when required to integrate visual feedback and internal prediction, suggesting a decline in their ability to flexibly update beliefs in response to external cues [127]. This aligns with a dynamical systems perspective in which decision-making emerges from the interplay between preparatory neural states (prior beliefs) [128] and real-time sensory evidence, modulated by precision estimation mechanisms akin to Kalman gain. Here, Mu and beta–gamma rhythms—linked to the “Brain as Observer” and “Brain as Controller”, respectively—dynamically modulate corticospinal excitability during movement [55,129,130,131]. Their disruption in FND may be addressed through mental imagery and external suggestions (for operant conditioning) [48,132]. The cerebellum, through its connections with the MPFC and thalamocortical circuits, supports Hidden State Transitions and Correction Steps [133]. Sensory deafferentiation in the context of FND refers to the disruption or abnormal processing of afferent sensory signals—particularly reafferent feedback (i.e., sensory input generated by one’s own movements) [41]. In FND, sensory deafferentiation arises not from peripheral nerve damage but from altered central processing. Maladaptive learning may lead the brain to ignore sensory feedback, modeled as a Kalman filter, where measurement noise is perceived as extremely high (R→∞), confidence in internal predictions is excessive (P(t∣t−1)→0), and the observation model fails to link sensory input to the internal state ($\hat{H}\to 0$). As a result, the Kalman gain K(t) approaches zero ($K(t)={P(t|t-1){\hat{H}}^{T}[\hat{H}P(t|t-1){\hat{H}}^{T}+R]}^{-1}\to 0$), preventing updates from sensory feedback and disrupting sensorimotor integration for motor control [41]. From a functional dystonia and tremor case observed during our usability study [51], we can simulate key sensorimotor disruptions computationally for goal-directed movement, r=1. The Matlab (Mathworks Inc. USA) code (https://github.com/Siagnos/ICRA2025 accessed on 10 April 2025) captured the core mechanisms of functional dystonia (FD)—disrupted prediction, sensory feedback, and motor control [134].

**Figure 11:**
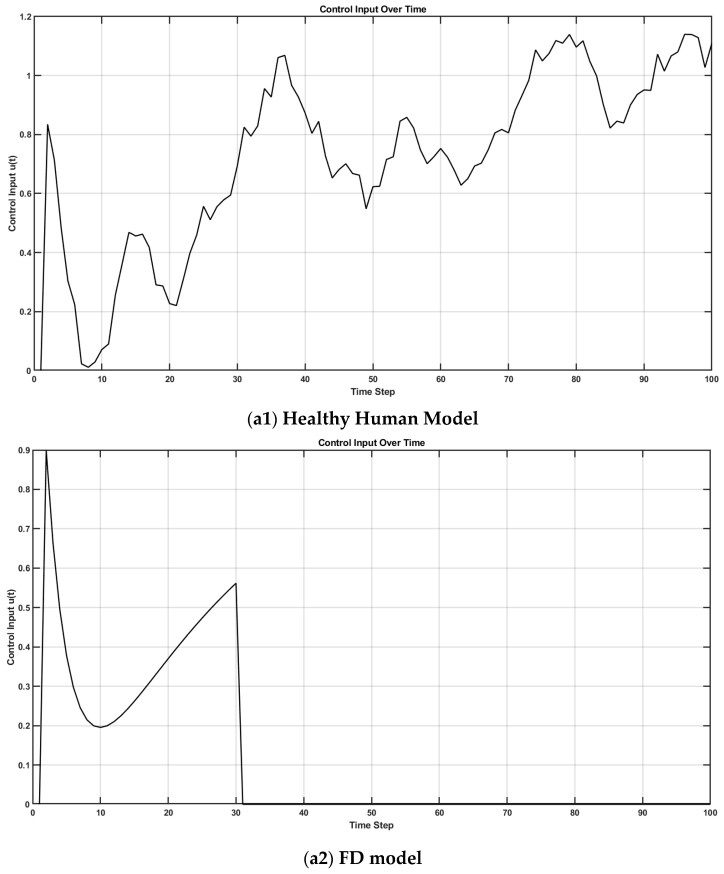

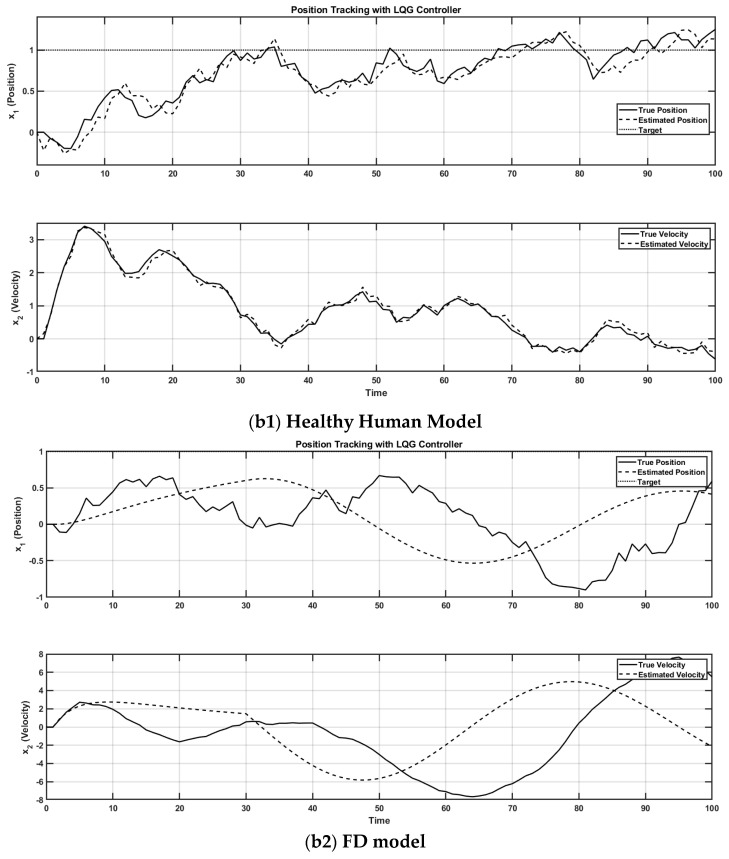
Healthy human model shows smooth and sustained control input (**a1**), accurate position tracking with close alignment between true and estimated states, and stable velocity dynamics (**b1**). In contrast, the functional dystonia (FD) model shows that control input rapidly diminishes and stops early (**a2**), leading to poor position tracking and disrupted oscillatory velocity patterns (**b2**), consistent with impaired motor execution and sensorimotor integration observed in FD.

**Figure 12:**
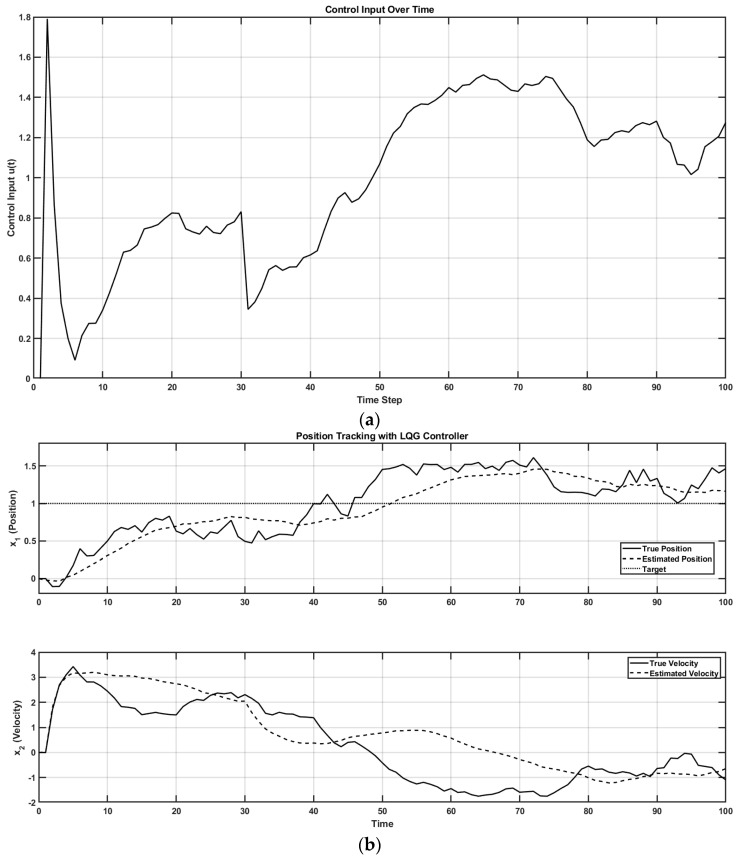
Adaptive haptic feedback restores motor control in an FD model by correcting early control cessation with robotic assistance—combined control input shown in (**a**). A predictive LQR controller for bottom-up robotic assistance and top-down suggestion-enhanced mixing gain in human improved position and velocity tracking (**b**).

Figure 11 shows the simulation of the Kalman filter and LQG control model to represent motor behavior in a healthy human versus FD model. It integrates a traditional LQG controller for goal-directed movement with an external adaptive haptic controller (modeled as a predictive LQR since “Virtual World” spring-damper model is known—see Figure 1), simulating XR-based assistance. To capture the dysfunctions seen in FD, the mixing gain is artificially reduced to simulate deafferentation or overconfident prediction, while motor imagery suggestion is modeled by amplifying this mixing gain. The system incorporates elements of effort–reward miscalibration, motor suppression, and involuntary movement using randomized effort thresholds, control suppression, and low-frequency motor command biases, respectively. The result is a hybrid system that blends control, assistance, and pathological interference, making it suitable for studying motor dysfunction in sensorimotor disorders like FD. Figure 12 illustrates that adaptive haptic exafference restores motor control in FD by using predictive LQR assistance (“Virtual World” spring-damper model is known—see Figure 1), while top-down suggestions enhance human state estimation through increased mixing gain. Indeed, suggestions have been shown to influence these processes by modulating agency and prediction through the cerebellar and SMA pathways [135,136,137,138,139], offering potential for therapeutic intervention in disorders of agency and motor control. 

Altered states of consciousness, such as those induced by meditation and hypnosis, modulate brain dynamics, e.g., Brechet et al. [140] demonstrated that intense meditation training significantly reshapes EEG microstates. Two novel microstate topographies emerged post meditation, accounting for nearly 50% of the variance in EEG data. These patterns are localized to brain areas involved in self-related multisensory experience, including the insula, supramarginal gyrus, and superior frontal gyrus. Notably, meditation disrupted the dominance of Microstate C—during internal processing (without suppression, just with increased awareness) likely subserved by the SCAN—suggesting a departure from the typically stable microstate architecture found in prior studies [141]. These findings support the hypothesis that meditation enhances internal processing without suppression, aligning with the increased self-awareness reported during hypnotic states [96]. Katayama et al. [141] investigated EEG microstate changes across stages of hypnosis—resting, light, deep hypnosis, and recovery. They found that during deep hypnosis, Microstates A and C increased in duration and time coverage, while Microstates B and D decreased. This transition is postulated to reflect a shift in cognitive dominance from executive control toward self-reflective internal processing without suppression. Recovery stages showed intermediate characteristics, highlighting a dynamic trajectory through altered states. Importantly, Katayama’s results suggest that deep hypnosis may enhance insight by amplifying “Brain as Observer” activity while dampening “Brain as Controller” functions. Comparisons across states showed that deep hypnosis shared traits with schizophrenia (e.g., hallucinations), while light hypnosis overlapped with meditation in promoting relaxation and awareness. This observation aligns with findings by Xu et al. [142], who explored self-projection and future thinking. Participants reported higher vividness and self-projection when imagining their Future Self compared to their Present Self. A functional dissociation emerged within the DMN: the anterior DMN (aDMN) was more active during Present Self-reflection (“Brain as Observer”), while the posterior DMN (pDMN) was more active during Future Self imagery or Projection (“Brain as Controller”). These subsystems may be selectively modulated through internal simulation and suggestion-based techniques, particularly in therapeutic contexts like self-hypnosis [41]. Within this broader context, the Kalman filter and LQG control framework offers a powerful model to explain how the brain learns, corrects errors, and adapts motor skills—see Table 3. In my proposed model, microstate analysis and enactment imagination [99] can be used alongside multimodal brain imaging to distinguish between novice and expert skill acquisition. 

Novices display variable, exploratory neural pathways, while experts show efficient transitions across the FPN, DMN, and SN during skill learning [109]. Here, the SMA plays a central integrative role, transforming internal motor intentions into precise executions, informed by external feedback including suggestions. For example, a steady decline in the strength of directed functional connectivity from the lateral prefrontal cortex to the SMA over the course of a 15-day training period was found [109]. During the initial “Cognitive stage” (Days 1–5), connectivity was high, reflecting strong top–down control from the LPFC as participants engaged in conscious, effortful learning. As training progressed into the “Associative stage” (Days 6–15), this connectivity gradually decreased, indicating reduced reliance on executive control and increased motor automation. By the retention phase, the LPFC→SMA influence remained low, suggesting that the skill has become internalized and more efficiently executed. This shift reflects the brain’s transition from effortful to automatic processing, a hallmark of sensorimotor learning. However, in FMD, this process breaks down—disrupted agency and impaired SMA signaling interfere with adaptive motor control [46]. This literature review of the Kalman filter and LQG control framework revealed how internal simulations and external suggestions during enactment imagination can be harnessed to recalibrate these pathways and restore voluntary control.

The SCAN offers a unifying framework for understanding mind–body aspects of how the brain integrates intention, planning, and movement [38]. It plays a central role in coordinating complex, full-body motor plans by drawing on body schema and task goals, allowing for anticipatory adjustments in posture, arousal, and attention. The SCAN also contributes to the SoA, helping bind intention with action and feedback, and may be disrupted in conditions like alien limb syndrome, FMD, or motor delusions. Functionally, SCAN connects motor control with cognitive and emotional processes, supporting tasks that demand the real-time integration of movement and mental focus—such as performing under stress. Its role in motor imagery and rehabilitation is especially notable, as engaging the SCAN during mental practice may enhance motor recovery by strengthening global mind-body action frameworks and enabling inter-limb learning. In HMI applications, leveraging the SCAN through kinesthetic imagery whole-body action preconditioning may yield more robust brain-based control signals. Overall, the SCAN bridges cognitive processes with motor execution, enriching our understanding of volitional movement, embodied cognition, and neurorehabilitation. Across neuroimaging studies, SCAN-related regions like the SMA, parietal cortex (IPL/TPJ), cerebellum, and prefrontal cortex emerge as central hubs for generating, monitoring, and attributing agency. Here, the Contingent Negative Variation (CNV) is postulated to reflect the scalp-level electrophysiological activity of the SCAN, with its amplitude and distribution corresponding to the engagement of SCAN-related regions—such as the SMA, midcingulate cortex, thalamus, and insula—during anticipatory, time-based, and goal-directed motor–cognitive preparation [143,144,145]. Specifically, CNV may index slow cortical potentials associated with increased cortical excitability [144], consistent with the SCAN’s role in integrating cognitive control, motor readiness, and internal timing mechanisms [145]. Then, using anodal transcranial direct current stimulation (tDCS) over the M1, we found increased theta-band EEG activity and a reduced slope in slow cortical potentials during self-initiated movement in healthy participants [146]. Our findings suggest that anodal tDCS modulates brain activity patterns linked to movement preparation, possibly by influencing decision-related neural processes involving the SCAN at the M1. Here, structural connectivity between the SMA/pre-SMA and parietal cortex via the superior longitudinal fasciculus (SLF), particularly its branches (SLF I and SLF II), further supports such integrated brain functions [147], motor intentionality [148], and the sense of agency [149,150]. Also, the transcranial electrical stimulation of angular gyrus–middle frontal gyrus connections, linked via the dorsal SLF II, may enhance the sense of agency. Similarly, targeting the supramarginal gyrus–inferior frontal gyrus via SLF III may support perception in complex tasks. In experts, the preSMA–LPFC coupling, supported by the extended frontal aslant tract, may underlie sequence learning and executive control in familiar tasks. Overall, optimally targeted transcranial electrical stimulation may boost predictive coding and efference copy signaling, aiding motor skill learning and neurorehabilitation [93,146,151,152], e.g., the transcranial electrical stimulation of the SCAN nodes of M1.

## Figures and Tables

**Table 1:** Significant computational models of predictive coding (perception–action coupling).

Authors (Year)	Model Name/Type	Core Assumptions	Computational Framework	Application Domain
Srinivasan, Laughlin, and Dubs (1982) [11]	Predictive coding (PC) in retina	Retina uses lateral inhibition to predict and cancel expected input (center–surround); reduces redundancy and noise.	Linear predictive coding (subtract prediction from signal)	Early vision (retinal encoding)
Mumford (1992) [14,15]	Cortical predictive coding (theory)	Hierarchical cortico-cortical loops: higher areas send top–down predictions; lower areas send unexplained residuals upward.	Theoretical Bayesian hierarchy (conceptual model of cortex)	Visual cortex (general cortical function)
Rao and Ballard (1999) [16]	Hierarchical PC in visual cortex	Higher-level visual units predict lower-level responses; only prediction errors propagate feedforward. Explains context effects in V1.	Hierarchical generative model with error-feedback learning (gradient descent on prediction error)	Visual perception (extra-classical RF effects, V1–V2 interactions)
Friston (2005) [17]	Variational free-energy PC (Bayesian brain)	Brain minimizes “free energy” (prediction error + complexity). Cortical areas carry approximate posteriors; prediction errors drive inference.	Variational Bayesian inference (free-energy minimization); hierarchical dynamic model	Multisensory cortical processing (unified perception theory)
Friston et al. (2010) [18]	Active inference (continuous)	Perception and action both minimize prediction errors. Motor commands realize predicted sensory states by reducing proprioceptive error.	Dynamic Bayesian network (state-space model) with action feedback; expected free-energy minimization for policy selection	Sensorimotor control (e.g., oculomotor reflexes, limb control)
Spratling (2008) [9]	Predictive coding with biased competition (PC/BC-DIM)	Cortical predictions modulate lower-level activity divisively; predictive coding integrated with attention (biased competition) mechanism.	Nonlinear neural network (divisive inhibition for error calculation); still error minimization principle	Visual attention and feature binding (cortical V1/V2 function)
Bastos et al. (2012) [19]	Canonical microcircuit for PC	Specific laminar circuit mapping: superficial pyramidal neurons carry prediction errors upward; deep pyramidal neurons send predictions downward.	Conceptual neural architecture (informed by anatomy and Dynamic Causal Modeling)	General cortical implementation (e.g., vision, audition microcircuits)
Lotter, Kreiman, and Cox (2017) [20]	PredNet (deep predictive coding network)	Each layer of a deep network predicts the next input and forwards only errors. Unsupervised learning via prediction of future sensory input (frames).	Deep convolutional recurrent network following predictive coding architecture (multiple layers of prediction and error units)	Visual sequence learning (video frame prediction, unsupervised vision, driving scenes)
Parr and Friston (2019) [21]	Active inference (discrete, Partially Observable Markov Decision Process)	Agent evaluates possible action sequences via expected free energy (combining predicted rewards and information gain); perception and decision-making unified.	Partially Observable Markov Decision Process with variational message passing (belief propagation for inference and planning)	Goal-directed behavior and planning (sequential decision-making, foraging tasks)
Rao, Gklezakos, and Sathish (2024) [22]	Active Predictive Coding (APC)	Unified hierarchical model learning both state dynamics and policies. Predictive coding used for compositional representation learning and multi-step planning.	Multi-level generative model with integrated policy networks; uses hypernetworks and deep learning techniques within a predictive coding loop	High-level perception and action (e.g., learning object–part hierarchies, long-horizon planning in AI agents)

**Table 2:** Comparison of network engagement during motor execution vs. motor imagery, highlighting the role of SCAN (inter-effector) regions and related circuitry.

Network Engagement	Motor Execution	Mental Simulation
Primary M1 effector zones	Strongly activated for the specific moving body part; large corticospinal output drives muscles.	Moderately activated (sub-threshold activity); increased excitability but output gated (no overt movement).
SCAN inter-effector zones	Active to integrate multi-limb coordination, posture, and link movement with goals. Assist in planning and adjusting movement (feedback integration).	Active to simulate the action plan and sense of movement. Likely more relative involvement since they generate the internal action representation without triggering muscles. Contribute to the feeling of “agency” and urge during imagination.
Sensory feedback (S1)	High actual feedback from proprioception, touch, etc., fed into primary sensory cortex S1; SCAN bridges to S1 (via “plis de passage”) to incorporate this feedback.	Little to no actual feedback (no movement), but predicted feedback may be internally generated. SCAN’s connection to BA3a (proprioceptive area) might simulate expected sensations.
Subcortical involvement	Strong involvement of basal ganglia and cerebellum for executing movement (initiating and refining motions); SCAN connects to these (putamen, cerebellum) to help orchestrate execution.	Basal ganglia engaged in suppressing actual movement (inhibition via striatal circuits) while still allowing the thalamocortical loop of the plan. Cerebellum active in simulating the dynamics of the imagined movement. SCAN connectivity to these regions supports running the “motor program” offline.
Cognitive network coupling	Moderate: cognitive control (dACC/insula) signals ensure the movement meets the goal, especially if adjustments or attention is needed. Some cognitive resources focus on external execution monitoring.	Strong: sustained engagement of prefrontal, attention, and imagery networks (visual or kinesthetic imagery recruits frontoparietal and occipital areas). SCAN nodes heavily coupled with cingulo-opercular network to maintain the intention in mind without output.

**Table 3:** Kalman filter framework and related brain circuitry.

Kalman Filter Component	Brain Regions	Function Alignment
State Representation	Posterior parietal cortex (PPC), motor cortex, and cerebellum	Encodes current estimated body state; integrates proprioceptive, visual signals.
Internal Models	Cerebellum (forward model) and premotor cortex (inverse model)	Predicts sensory outcomes from efference copy of motor command.
Error Computation	Cerebellum and anterior cingulate cortex (ACC)	Computes prediction errors between expected and actual sensory input.
Gain Adjustment	Basal ganglia and thalamus	Adjusts how much weight to give to prediction vs. sensory feedback (Kalman gain).
Control Update	Motor cortex and supplementary motor area (SMA)	Updates motor plans based on corrected state estimates.
Cognitive Modulation	Prefrontal cortex (PFC) and ACC	Modulates attention, strategy, and confidence in the internal model.

## Data Availability

The MATLAB code used for the simulations in this paper is provided at https://github.com/Siagnos/ICRA2025 accessed on 10 April 2025.

## Abbreviations

The following abbreviations are used in this manuscript:

SoA	Sense of agency
HMI	Human–machine interface
XR	eXtended Reality
VR	Virtual reality
FND	Functional neurological disorder
EPH	Equilibrium Point Hypothesis
fMRI	Functional Magnetic Resonance Imaging
ERP	Event-Related Potential
DTI	Diffusion Tensor Imaging
SMA	Supplementary motor area
M1	Primary motor cortex
PFC	Prefrontal cortex
IPL	Inferior parietal lobule
TPJ	Temporoparietal Junction
DMN	Default mode network
FPN	Frontoparietal network
CON	Cingulo-opercular network
SN	Salience network
aDMN	Anterior default mode network
pDMN	Posterior default mode network
LQG	Linear–Quadratic–Gaussian
DT	Dynamical Systems Theory
tES	Transcranial electrical stimulation
AG	Angular gyrus
MFG	Middle frontal gyrus
SMG	Supramarginal gyrus
IFG	Inferior frontal gyrus
LPFC	Left prefrontal cortex
RPFC	Right prefrontal cortex
LPMC	Left premotor cortex
exFAT	Extended frontal aslant tract
fNIRS	Functional near-infrared spectroscopy
EEG	Electroencephalography
MS	Microstate
PPIE	Patient and Public Involvement and Engagement
CFR	Counterfactual regret minimization
GLM	General Linear Model
GEV	Global Explained Variance
HRX-1	Haptic Rehabilitation eXtended robot 1 (HumanRobotix London)
BOLD	Blood Oxygen Level Dependent (implied in neuroimaging context)
A, B, C, D, E, F, C′	EEG microstate classes
